# Differentiation of ncRNAs from small mRNAs in *Escherichia coli* O157:H7 EDL933 (EHEC) by combined RNAseq and RIBOseq – *ryhB* encodes the regulatory RNA RyhB and a peptide, RyhP

**DOI:** 10.1186/s12864-017-3586-9

**Published:** 2017-02-28

**Authors:** Klaus Neuhaus, Richard Landstorfer, Svenja Simon, Steffen Schober, Patrick R. Wright, Cameron Smith, Rolf Backofen, Romy Wecko, Daniel A. Keim, Siegfried Scherer

**Affiliations:** 1 Lehrstuhl für Mikrobielle Ökologie, Wissenschaftszentrum Weihenstephan, Technische Universität München, Weihenstephaner Berg 3, D-85354 Freising, Germany; 2 Core Facility Microbiome/NGS, ZIEL Institute for Food & Health, Weihenstephaner Berg 3, D-85354 Freising, Germany; 3 Informatik und Informationswissenschaft, Universität Konstanz, D-78457 Konstanz, Germany; 4 Institut für Nachrichtentechnik, Universität Ulm, Albert-Einstein-Allee 43, D-89081 Ulm, Germany; 5 Bioinformatics Group, Department of Computer Science and BIOSS Centre for Biological Signaling Studies, Cluster of Excellence, University of Freiburg, D-79110 Freiburg, Germany

## Abstract

**Background:**

While NGS allows rapid global detection of transcripts, it remains difficult to distinguish ncRNAs from short mRNAs. To detect potentially translated RNAs, we developed an improved protocol for bacterial ribosomal footprinting (RIBOseq). This allowed distinguishing ncRNA from mRNA in EHEC. A high ratio of ribosomal footprints per transcript (ribosomal coverage value, RCV) is expected to indicate a translated RNA, while a low RCV should point to a non-translated RNA.

**Results:**

Based on their low RCV, 150 novel non-translated EHEC transcripts were identified as putative ncRNAs, representing both antisense and intergenic transcripts, 74 of which had expressed homologs in *E. coli* MG1655. Bioinformatics analysis predicted statistically significant target regulons for 15 of the intergenic transcripts; experimental analysis revealed 4-fold or higher differential expression of 46 novel ncRNA in different growth media. Out of 329 annotated EHEC ncRNAs, 52 showed an RCV similar to protein-coding genes, of those, 16 had RIBOseq patterns matching annotated genes in other enterobacteriaceae, and 11 seem to possess a Shine-Dalgarno sequence, suggesting that such ncRNAs may encode small proteins instead of being solely non-coding. To support that the RIBOseq signals are reflecting translation, we tested the ribosomal-footprint covered ORF of *ryhB* and found a phenotype for the encoded peptide in iron-limiting condition.

**Conclusion:**

Determination of the RCV is a useful approach for a rapid first-step differentiation between bacterial ncRNAs and small mRNAs. Further, many known ncRNAs may encode proteins as well.

**Electronic supplementary material:**

The online version of this article (doi:10.1186/s12864-017-3586-9) contains supplementary material, which is available to authorized users.

## Background

Bacterial RNA molecules consist of non-coding RNAs (ncRNAs including rRNAs and tRNAs), and protein-coding mRNAs. ncRNAs are encoded either in *cis* or in *trans* of coding genes and their size ranges from 50–500 nt [1, 2]. *Cis*-encoded ncRNA templates are localized opposite to the gene to be regulated and, accordingly, have full complementarity to the mRNA. Their expression leads to a negative or positive impact on the expression of the regulated gene [3–5]. This type of gene regulation has been exploited in applied molecular biology [6]. However, only few experimentally verified *cis*-encoded ncRNAs exist, in contrast to *trans*-encoded ncRNAs. *Trans*-encoded ncRNAs are usually found in intergenic regions and have a limited complementarity to the regulated gene. Recent research has led to the view that *trans*-encoded ncRNAs are involved in the regulation of almost all bacterial metabolic pathways (see [7], and references therein).

The number of annotated ncRNAs known from different bacterial species is rapidly increasing. For instance, 329 ncRNAs are annotated for *E. coli* O157:H7 str. EDL933 [2]. Around 80 of them have been experimentally verified in *E. coli* [8]. Numerous bioinformatic studies on *E. coli* K12 and other bacterial species predicted the number of ncRNAs to range between 100 and 1000 (e.g. [9–11]). As *E. coli* O157:H7 strain EDL933 (EHEC) contains a core genome of 4.1 Mb which is well conserved among all *E. coli* strains [12], many similar or identical ncRNAs are assumed to exist in EHEC.

In the past, ncRNAs have been predicted by different bioinformatics methods (see [13] for a review about ncRNA detection in bacteria). A commonly used tool in ncRNA-prediction is RNAz, which has been used to predict ncRNAs in *Bordetella pertussis* [14], *Streptomyces coelicolor* [15] and others. However, any such studies require experimental verification [13] of which next-generation sequencing is of prime interest for this task.

While experimental large scale screenings for ncRNAs, especially strand-specific transcriptome sequencing using NGS, are becoming more and more important (e.g. [16–18]), it is not possible to determine whether a transcript is translated, based solely on RNAseq (see, e.g. [19]). In order to distinguish “true” ncRNAs from translated short mRNAs, we modified the ribosomal profiling approach developed by Ingolia et al. for yeast [20] and applied this technique to *E. coli* O157:H7 strain EDL933. Ribosomal profiling, which is also termed ribosomal footprinting or RIBOseq, detects RNAs which are covered by ribosomes and which are, therefore, assumed to be involved in the process of translation. The RNA population which is covered by ribosomes is termed “translatome” [21] and bioinformatics tools are now available to analyze these novel data [22]. Combined with strand-specific RNA-sequencing, we suggest that this approach provides additional evidence to distinguish between non-coding RNAs and RNAs covered by ribosomes.

In the past, RNAs have been found which function as ncRNA (i.e. having a function as RNA molecule not based on encoding a peptide chain) and, at the same time, as mRNA (i.e. encoding a peptide chain). Therefore, those RNAs were either termed dual-functioning RNAs (dfRNAs [23]) or coding non-coding RNAs (cncRNAs [24]). The former name is now used for RNAs with any two different functions (e.g., base-pairing and protein binding [25]), the latter describes the fact that the DNA-encoded entity functions on the level of RNA (hence, non-coding) and additionally on the level of an peptide (i.e. coding). Less than ten examples of cncRNAs are known from prokaryotes, e.g., RNAIII, SgrS, SR1, PhrS, *gdpS*, *irvA*, and others [23, 24, 26, 27].

## Methods

### Microbial strain

Strain *E. coli* O157:H7 EDL933 was obtained from the Collection l’Institute de Pasteur (Paris) under the collection number CIP 106327 (= WS4202, Weihenstephan Microbial Strain Collection) and was used in all experiments. The strain was originally isolated from raw hamburger meat, first described in 1983 [28], originally sequenced in 2001 [12] and its sequence improved recently [29]. The genome of WS4202 was re-sequenced by us to check for laboratory derived changes (GenBank accession CP012802).

### RIBOseq

Ribosomal footprinting was conducted according to Ingolia et al. [20], but was adapted to sequence bacterial footprints using strand-specific libraries obtained with the TruSeq Small RNA Sample Preparation Kit (Illumina, USA). Cells were grown in ten-fold diluted lysogeny broth (LB; 10 g/L peptone, 5 g/L yeast extract, 10 g/L NaCl) with shaking at 180 rpm. At the transition from late exponential to early stationary phase the cultures were supplemented with 170 μg/mL chloramphenicol to stall the ribosomes (about 6-times above the concentration at which trans-translation occurs [30]). After two minutes, cells were harvested by centrifugation at 6000 × *g* for 3 min at 4 °C. Pellets were resuspended in lysis buffer (20 mM Tris-Cl at pH8, 140 mM KCl, 1.5 mM MgCl_2_, 170 μg/mL chloramphenicol, 1% *v/v* NP40; 1.5 mL per initial liter of culture) and the suspension was dripped into liquid nitrogen and stored at −80 °C. The cells were ground with pestle and mortar in liquid nitrogen and 2 g sterile sand for about 20 min. The powder was thawed on ice and centrifuged twice, first at 3000 × *g* at 4 °C for 5 min and next at 20,000 × *g* at 4 °C for 10 min. The supernatant was saved and A_260nm_ determined. After dilution to an A_260nm_ of 200, RNase I (Ambion AM2294) was added to the sample to a final concentration of 3 U/μL and the sample was gently rotated at room temperature (RT) for 1 h. Remaining intact ribosomes with protected mRNA-fragments (footprints) were enriched by gradient centrifugation. A sucrose gradient was prepared in gradient buffer (20 mM Tris-Cl at pH 8, 140 mM KCl, 5 mM MgCl_2_, 170 μg/mL chloramphenicol, 0.5 mM DTT, 0.013% SYBR Gold). Nine different sucrose concentrations were prepared in 5% (*w/v*) steps ranging from 10 to 50% and 1.5 mL of each concentration was loaded to a centrifuge tube. Five hundred μL of the crude ribosome sample were loaded onto each gradient tube and centrifuged at 104,000 × *g* at 4 °C for 3 h. The layer containing the ribosomes was visualized using UV-light and the tube was pierced at the bottom to slowly release the gradient and the band containing intact 70S ribosomes was collected. To ensure that RNA which is not protected by ribosomes is fully digested, and to get a highly enriched ribosomal fraction, the procedure of RNase-digestion and gradient centrifugation was repeated: The ribosomal fraction was diluted 1:1 with gradient buffer (without SYBR Gold and sucrose) and was loaded on a sucrose gradient without the 10% sucrose layer. After centrifugation, complete 70S ribosomes were collected by slowly releasing the gradient as described above and frozen in liquid nitrogen. To obtain the protected ribosomal footprints, 1 mL Trizol was added to 200 μL of the ribosome suspension following the manual for Trizol extraction of RNA (life technologies, USA). The final footprint-RNA pellet was dissolved in RNase free water. To ensure no carry-over of genomic DNA fragments, DNase treatment was performed using the TURBO DNA-*free* Kit (Applied Biosystems, USA) according to the manual. For footprint size-selection, the crude RNA-preparation was loaded to a 15% denaturing polyacrylamide gel. An oligonucleotide of 28 bp was used as a marker which is about the size of a ribosomal footprint [31, 32]. After staining with SYBR Gold, the region of about 28 nt was excised from the gel. The RNA was extracted from the gel slice as described [20]. Results of pilot experiments showed that RNase I cuts the 5′ ends of the 16S rRNA producing a fragment of about the size expected for the footprints, contributing about 50% to the size-selected RNA fragments after sequencing. For this reason, these fragments were removed with oligonucleotides complementary to the 5′-end of the 16S rRNA using the MICROBExpress bacterial mRNA enrichment kit (life technologies, USA) following the manual. Furthermore, true footprints were found to be shorter than expected (see Results). Enriched footprint-RNAs were dephosphorylated using Antarctic phosphatase (10 units per 300 ng RNA, supplemented with 10 units Superase, 37 °C for 30 min). Footprints were recovered using the miRNeasy Mini Kit (Qiagen, Germany). Subsequent phosphorylation was carried out using T4 polynucleotide kinase (20 units supplemented with 10 units Superase, 37 °C for 60 min) and cleaned using the miRNeasy Mini Kit as before. Finally, the entire sample was processed with the TruSeq Small RNA Sample Preparation Kit (Illumina) according to the manual, using 11 PCR cycles, and was sequenced on an Illumina MiSeq.

### Transcriptome sequencing

The same cultures used for ribosomal footprinting were also used for transcriptome sequencing (i.e., strand specific RNAseq). Fifty μL of the diluted cell extract with an A_260nm_ of 200 units (see above) were added to one 1 mL of Trizol and total RNA was isolated. Since 90–95% of the total RNA consists of ribosomal RNA [33], the Ribominus Transcriptome Isolation Kit (Yeast and Bacteria, Invitrogen, USA) was applied according to the manual and the RNA was precipitated with the help of glycogen and two volumes 100% ethanol. DNase treatment was performed as described above. One μg RNA was fragmented as described [34] and the RNA-fragments were precipitated with glycogen and 2.5 volumes 100% ethanol. For sequencing on an Illumina MiSeq, the fragments were resuspended in 25 μL RNase free water and further processed like the cleaned footprint-RNAs (see above).

### Northern blots

RNA was isolated in the same manner and under the same conditions as for the NGS experiments. Northern blots were performed using the DIG Northern Starter kit (Roche, Switzerland). Primers to generate DIG (digoxygenin) labeled probes are listed in Additional file 1: Table S1. For preparation of the probes, electroblotting, crosslinking, hybridization and detection, the manufacturer’s protocol was followed, except that electroblotting was performed using polyacrylamide gels and that for crosslinking EDC (1-ethyl-3-(3-dimethylaminopropyl) carbodiimide) was used [35]. After exposure to CDP-Star (included in the DIG Northern Starter kit), luminescence activity of the hybridized probes was measured using an In-Vivo Imaging System (PerkinElmer, USA).

### Competitive growth assays for the overexpression phenotype of RyhP

For the production of the peptide RyhP encoded in RyhB, two versions of the corresponding ORF (named P1 and P2) were cloned onto pBAD/*Myc*-His C (Invitrogen). Similarly, two versions of this ORF with either the second or the third codon changed into stop codons to terminate translation were used as negative controls (named T2 and T3). For cloning, primer pairs (for primer see Additional file 1: Table S1) were hybridized forming RyhP-coding dsDNA fragments. The pBAD was opened by *Nco*I and *Bgl*II in restriction buffer NEB3.1 (NEB) and was subsequently column cleaned (Genelute PCR Clean-Up Kit, Sigma-Aldrich). RyhP-DNA fragments and pBAD were ligated (T4 ligase, NEB) and transformed in *E. coli* TOP10. After sequencing (eurofins), verified plasmids were transformed in *E. coli* O157:H7 EDL933. EHEC strains (containing either P1, P2, T2 or T3) were grown overnight in LB medium with a final concentration of 120 μg/ml ampicillin. The cell was density measured and both strains were mixed 50:50. Minimal Medium (MM) M9 without any iron added [36], but supplemented with a final concentration of 120 μg/ml ampicillin and 0.2% arabinose (for induction), was inoculated 1:1000 using the mixture and incubated 24 h at 37 °C with shaking at 150 rpm. Of both, the initial mixture and of the MM-culture, the plasmids were isolated and Sanger sequenced using the primer pBAD-C-R. The peak heights of the two nucleotides changed to form the stop codon in T2 or T3 were measured in comparison to the P variants, and the mean CI was calculated according to CI = (T(out) · P(in))/(P(out) · T(in)) [37] of P1 against, T2, P1 against T3 and P2 against T3. Given are mean and the standard deviations of three biological independent experiments.

### Bioinformatics procedures

#### NGS mapping and evaluation

Raw data were deposited at the Gene Expression Omnibus [GEO: GSE94984]. Illumina output files (FASTQ files in Illumina format) were converted to plain FASTQ using FastQ Groomer [38] in Galaxy [38, 39]. The FASTQ files were mapped to the reference genome (NC_002655) using Bowtie2 [40] with default settings, except for a changed seed length of 19 nt and zero mismatches permitted within the seed in the Illumina data due to the short length of the footprints. Visualization of the data was carried out using our own NGS-Viewer [41] or BamView [42] implemented in Artemis 15.0.0 [43].

The number of reads was normalized to reads per kilobase per million mapped reads (RPKM) [44]. Using this method, the number of reads is normalized both with respect to the sequencing depth and the length of a given transcript. For determination of counts and RPKM values, BAM files were imported into R (R Development Team [45]) using Rsamtools [46]. For further processing, the Bioconductor [47] packages GenomicRanges [48] and IRanges were used [49]. The locations of the 16S rRNA and 23S rRNA are given by the RNT file from RefSeq [50]. findOverlaps of IRanges [49] was used to determine the remaining reads overlapping a 16S or 23S rRNA gene on the same strand. Reads from these rRNA-genes were excluded from further analysis as most rRNA had been removed using the Ribominus kit, as described above. countOverlaps can also determine the number of reads overlapping a gene on the same strand (counts). Using these counts, RPKM values were generated. For the value “million mapped reads”, the number of reads mapped to the genome, less the remaining reads overlapping a 16S or 23S rRNA gene, were used. Pearson correlation was calculated using Excel and Spearman rank correlation according to Wessa [51].

#### RCV thresholds

To distinguish between translated and non-translated for a given RNA, the ribosomal coverage value (i.e., reads of ribosomal footprints per reads of mRNA) was examined [52]. A negative control set contains the RCVs of tRNAs (“untranslated”). Sixteen phage encoded tRNAs, one tRNA annotated as a pseudogene, and one tRNA containing less than 20 reads in the combined transcriptome data set were disregarded since phage tRNAs sometimes have unusual properties [53, 54]. The RCVs of the tRNAs were transformed to ln(RCV), abbreviated LRCV. A density function $f\overset{}{\hat{\mkern6mu} }$_LRCV-tRNA_(x), with x = LRCV, was estimated by a kernel density estimation with Gaussian kernels and bandwidth selection according to Scott’s rule [55], furthermore a normal distribution was fitted as well for comparison. This was also conducted for the annotated genes (i.e., “translated” set), excluding zero RCVs (261 genes). To test the hypothesis “the RCV of the RNA belongs to the tRNA distribution”, we used the estimated tRNA LRCV distribution to compute a *P* value for an observed ncRNA with LRCV x as

$$ Pval\ (x)={\displaystyle {\int}_x^{+\infty }{\mathrm{f}}_{\mathrm{LRCV}\hbox{-} \mathrm{tRNA}}\ (x) dx,} $$

where we numerically evaluate the density function. For example, the hypothesis will be rejected for α = 0.05 for any x ≥ −1.816817 which corresponds to an RCV of 0.162542. Similar for α = 0.01 we obtain an RCV of 0.354859. For α = 0.05 we reject 52 of 115 annotated ncRNAs to be not translated, and for α = 0.01 we reject 63.

Since the interpretation of the results depends on the assumed distribution, we also used, at least for tRNAs, a fit of the normal distribution. The tails of the normal distribution tend to zero faster than before, which results in different *P* values. For example, for α = 0.05 a corresponding RCV of 0.646079 is obtained and for α = 0.01 the bound for the RCV is 0.928702. However, the normal distribution has no good fit (not shown) and is henceforth excluded.

In a similar way as for the tRNAs, we can use the gene distribution to test the hypothesis “the RCV of the RNA belongs to the mRNA distribution” by using the RCV of all annotated genes (aORFs) as a negative control set. In this case, the *P* value is computed by

$$ Pval\ (x)={\displaystyle {\int}_{-\infty}^x{\mathrm{f}}_{\mathrm{LRCV}\hbox{-} \mathrm{aORF}}(x) dx.} $$

For the latter function, we obtained the bounds 0.532837 and 0.197320 for α = 0.05 and α = 0.01, respectively. Thus, all RNAs above those values might be considered mRNAs.

#### Examination of known and novel ncRNAs

*Escherichia coli* O157:H7 EDL933 (genbank accession AE005174) contains 329 known ncRNAs (Rfam database, April, 30^th^ 2014 [56]). All ncRNAs which should naturally have ribosomal footprints (e.g., are leader peptides, riboswitches (several contain a translatable ORF [57]), occur within genes on the same strand, or tmRNA) were excluded from the analysis, as well as rRNAs and tRNAs. Thus, the excluded RNAs are 5S_rRNA (8x), ALIL (19x), Alpha_RBS, C4, Cobalamin, *cspA* (4x), DnaX, FMN, greA, His_leader, IS009 (3x), IS102 (2x), iscRS, isrC (2x), isrK (2x), JUMPstart (3x), Lambda_thermo (2x), Leu_leader, Lysine, Mg_sensor, mini-ykkC, MOCO_RNA_motif, nuoG, Phe_leader (2x), PK-G12rRNA (7x), QUAD_2, rimP, rncO, rnk_leader, rne5, ROSE_2, S15, SECIS (3x), SgrS, ssrA (tmRNA), sok (10x), SSU_rRNA_archaea (14x), STnc40, STnc50, STnc370, t44/ttf, Thr_leader, TPP (3x), tRNAs (99x), tRNA-Sec, Trp_leader, and yybP-ykoY. The remaining 116 RNAs were grouped in translated, non-translated and undecided according to their RCV. Translated ncRNAs were three-frame translated and proteins sequences were searched against the non-redundant database “nr” of genbank using blastp [58]. Cases in which the ORFs of the ncRNA generated a single hit to the database were excluded since a false annotation of the hit is likely for those.

In order to provide an initial *in silico* characterization of the putative function for the novel intergenically-encoded ncRNAs, we used CopraRNA [59, 60] and examined the functional enrichments returned for the predictions. CopraRNA was called with default parameters for each set of putative ncRNA homologs. To find ncRNA homologs for the CopraRNA prediction, GotohScan (v1.3 stable) [61] was run with an e value threshold of 10^−2^ against the set of genomes listed in the Additional file 2: Table S2. The highest scoring homolog (i.e. having the lowest e value) for each organism was retained, if more than one GotohScan hit was present.

#### Ka/Ks ratio

The most likely ORF encoding a peptide was chosen according to the RIBOseq data. Homologs were searched using NCBI Web BLAST in the database nr using blastn. Hits with the highest e value but still achieving 100% coverage and displaying no gaps in the alignment were chosen (Additional file 3: Table S3). Gene pairs were examined using the KaKs_Calculator 2.0 [62] providing a number of algorithms which are compared and evaluated.

#### Shine-Dalgarno prediction

For any novel ncRNA with a significant blastp hit (e value ≤ 10^−3^, see above), a start codon (ATG, GTG, TTG) of the respective frame was searched closest to the start position of the ncRNA (except *sgrS* for which the start codon position is known, but ATG in *E. coli* K12 corresponds to ATT in EHEC, a rare but possible start codon; see Discussion). The maximum distance allowed between the ncRNA start coordinate and proposed start codon was ±30 bp. The region upstream of the putative start codon was examined for the presence of a Shine-Dalgarno sequence (optimum taAGGAGGt) according to [63] and [64]. A Shine-Dalgarno motif was assumed to be present at a Δ*G°* threshold of ≤ −2.9 kcal/mol (according to [63]) to allow weak Shine-Dalgarno sequences to be reported since even leaderless mRNAs exist [65].

For global examinations, we used PRODIGAL bins of the Shine-Dalgarno sequence and their distance to the start codon (Additional file 4: File S1) according to Hyatt et al. [66]. Bins without genes were omitted, and bins containing less than 100 genes were combined to superbins: S0, S2-3-4, S6, S7-8-9-12, S13, S14-15, S16, S18-19-20, S22, and S23-24-26-27 containing 629, 115, 116, 133, 1095, 664, 1191, 145, 687, and 327 genes, respectively.

## Results and discussion

### Sequencing statistics and footprint size

Two biologically independent replicates were used to assay reproducibility (Additional file 5: Figure S1). The numbers of footprint reads per gene of both RIBOseq replicates have a Pearson correlation of 0.86 and a Spearman rank correlation of 0.92, which was found to be slightly less compared to other NGS experiments [17, 67]. Nevertheless, the data sets were combined to increase the overall sequencing depth. In summary, 32.0 million transcriptome reads and 20.6 million translatome reads could be mapped to the EHEC genome (NC_002655; see Additional file 6: Table S4). Interestingly, the percentage of tRNA, an RNA species not translated, in both experiments was quite different. In the transcriptome, tRNAs contributed 31% of the library, whereas in the footprint libraries, tRNAs contributed only 0.3%. Such a difference is expected, since in the transcriptome sequencing, the tRNAs are processed together with the total RNA isolated. In contrast, in translatome sequencing, only translated RNAs are sequenced since the RNase digestion will destroy any RNA outside the ribosomes, including most tRNAs. However, some tRNAs might be trapped in the ribosomes and are recorded despite the RNase treatment. Thus, we reasoned that tRNAs would represent the best maximum background value for any carry-over of a non-translated RNA in the translatome sequencing.

The number of nucleotides which are protected by the ribosomes, i.e., the size of the footprints, was reported to be 28 nt in prokaryotes as well as in eukaryotes [20, 31, 32, 34, 68, 69]. Additionally, other studies using ribosome profiling in eukaryotes were able to determine the ribosome position of the footprints at sub-codon resolution (e.g. [70, 71]). The situation is quite different in bacteria: In one of the first studies in bacteria, Li et al. [72] determined the footprint size to range between 25 and 40 nt. Based on these results, O’Connor et al. [73] suggested that the footprint size may vary due to different progression rates of the ribosome. However, the enzyme used to obtain the bacterial ribosomal footprints in these studies was micrococcal nuclease which is known to prefer sites rich in adenylate, deoxyadenylate or thymidylate, which explains the varying length of the footprints [72]. In our study, after sequencing *E. coli* ribosomal footprints, the major peak of fragment sizes was observed at 23 nt, even despite the size-selection targeting 28 nt. We believe that RNase I, which we used, is a better choice [74, 75]. We also tested a number of commercially available RNases and mixtures of endo- and exo-cutting enzymes and received a consistent footprint size of about 23 nt and not 28 nt (unpublished data). The observed value of 23 nt may be explained by the different size of prokaryotic and eukaryotic ribosomes. Klinge et al. [76] estimated the mass of ribosomes to be 3.3 MDa for the eukaryotic and 2.5 MDa for prokaryotic, respectively. Assuming a roughly proportional scaling between the mass of the ribosome and its diameter suggest a bacterial footprint size of about 23 nt.

### Putative novel ncRNAs with low ribosomal coverage

The ribosome coverage value (RCV) gives the ratio of RPKM footprints over RPKM transcriptome. ncRNAs should have low RCVs. The RCV is similar to the “translational efficiency” applied for eukaryotes [77] to determine the translatability of a given mRNA. The RCV varied between zero (for 261 annotated genes) and a maximum value of nearly 39 for an annotated gene. Low or zero RCVs for annotated genes can be explained by the internal status of the cells controlling translation independent of transcription. For instance, some mRNAs are blocked by riboswitches or bound by ncRNA (e.g. [78]). We examined the genes with zero reads in some detail. This group contains about 3-times more phage associated genes compared to all genes (36% versus 13%). The genes are shorter compared to all (about half the size) and a larger fraction is annotated as hypothetical (50% compared to 30% in the annotation NC_002655). We looked for transcription under any of 11 different growth conditions [17] and found transcription for less than 20% of those genes under any condition. However, the other genes might be activated in specific circumstances not tested yet. This is corroborated by our findings that some genes were induced when EHEC was grown in co-culture with amoeba (unpublished results), but are not activated in any other condition of the published data set [17].

To analyze the data for novel ncRNAs, the transcriptome data was analyzed for contiguous transcription patterns (no gaps allowed) containing at least 20 transcriptome reads which do not correspond to an annotated gene (i.e., in a distance of more than 100 nt to a same-strand annotated ORF of a gene). Start and end of the novel ncRNAs were defined as the first and last nt of the contiguous read pattern. The chosen value of 20 reads was applied independently of any length restriction. For a 100-bp transcript in our dataset this approximately corresponds to an RPKM of 20, which is about 200-times above background level for transcriptome sequencing [17].

**Fig. 1:**
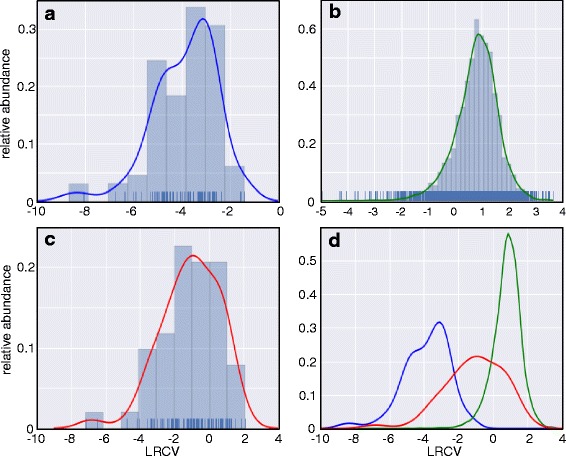
Logarithmic (ln) ribosomal coverage (LRCV) of tRNAs, annotated genes, annotated ncRNAs and a merger of the former. **a** Histogram of the LRCVs (X-axis) of the tRNAs together with either the estimated density function (*blue curve*). The density of the individual tRNAs is shown as little *blue* bars on top of the X-axis. **b** LRCV histogram as before, but of the annotated genes and their estimated density function (*green*). **c** LRCV histogram as before, but of the known ncRNAs (see Table 1) together with their estimated density function (*red*). **d** A combination of the estimated density functions for the tRNAs (*blue*), the annotated genes (*green*) and the ncRNAs (*red*) of the former panels, shown a substantial overlap between the annotated genes and the ncRNAs supposedly non-coding

Each novel transcript was analyzed for its RCV to determine whether it is potentially translated. As a negative control, we chose tRNAs which have RCVs in a range between 0.000173 and 0.094843. While the RCVs are small for tRNAs, the ratio between the highest and lowest RCV of the tRNAs is about 500-fold. We surmised that tRNA abundance might correlate either to the RCV or to the codon usage of EHEC (which correlates with tRNA abundance). However, no relationship was found (not shown) and the reasons for the difference in RCV remain unknown. For convenience, the RCV is shown as ln(RCV) (=LRCV) in Fig. 1. Figure 1a shows a histogram of the LRCV of tRNAs together with an estimated density function $\hat{f}$_LRCV_ (x) obtained by a kernel density estimation (blue line). Next, the LRCV distribution of the annotated genes is shown in Fig. 1b (green line). Finally, Fig. 1c shows the LRCV of all annotated ncRNAs (red line; less those known to be translated; see Table 1). To determine, whether the RCV of a given RNA belongs either to the tRNA distribution group or the gene distribution group, we determined the lower and upper limit of the RCV corresponding to a probability of error of 99% (α = 0.01), respectively (see Methods). Below the RCV threshold 0.197 a transcript is considered to be untranslated and above 0.355 it is considered to be a candidate for translation. Thus, a transcript is qualified as a putative novel ncRNA only, if its RCV was below the lower threshold.

**Table 1:** Transcriptome and translatome profiles of 115 ncRNAs known from *E. coli* O157:H7 EDL933

Name	Start position in the genome	Length	Strand	Number of transcriptome reads	Number of footprint reads	RPKM transcriptome	RPKM footprints	RCV	*P* value*	Northern Blot/Shine Dalgarno
DicF_1/Z1327	1255006	52	-	2	7	2	16	8.00	1.55E-11	
STnc70	719959	94	+	47	141	28	182	6.50	4.83E-11	
RyhB	4367464	65	-	92	192	80	359	4.49	1.77E-09	
OmrA-B_2	3766084	82	-	504	844	348	1251	3.59	1.47E-08	
OrzO-P_2	2954314	76	+	5057	8198	3764	13114	3.48	1.97E-08	taaagtggt
STnc100_10	2995675	210	-	496	742	134	430	3.21	4.12E-08	tatgggata
STnc550	2412748	391	-	533	779	77	242	3.14	4.96E-08	caaatagtg
RtT_3	1824178	132	-	22	28	9	26	2.89	1.03E-07	
RprA	2445280	108	+	568	745	297	839	2.82	1.25E-07	
STnc180	2250970	203	-	1225	1534	341	919	2.70	1.86E-07	caagcgggg
GadY	4474223	114	+	213	248	106	264	2.49	3.55E-07	
STnc630	5216481	166	+	502	572	171	419	2.45	4.05E-07	aacggagga
STnc100_1	902843	159	+	1046	1049	372	802	2.16	1.11E-06	
CyaR_RyeE	2912765	86	+	16620	16668	10932	23563	2.16	1.11E-06	
sroE	3426663	92	-	64	63	39	83	2.13	1.22E-06	
Z6077/DicF_4	2325956	52	+	118	112	128	262	2.05	1.64E-06	
C0299	1763522	79	+	1	1	1	2	2.00	1.96E-06	
RtT_2	1824000	132	-	3	2	1	2	2.00	1.96E-06	gaccaaggt
QUAD_7	4002118	150	-	859	791	324	641	1.98	2.12E-06	
tpke11	14107	78	+	59	51	43	79	1.84	3.64E-06	
STnc100_5	1866224	209	+	5038	4068	1364	2366	1.73	5.48E-06	
MicA	3606250	72	+	1500	1180	1178	1992	1.69	6.54E-06	
STnc100_3	1353605	206	+	2403	1688	660	996	1.51	1.41E-05	
sroD	2565135	86	-	94	65	62	92	1.48	1.58E-05	
MicC	2113860	122	-	43	29	20	29	1.45	1.83E-05	
frnS	2168565	118	-	175	106	84	109	1.30	3.70E-05	tcagggcaa
OmrA-B_1	3765887	88	-	696	380	447	525	1.17	6.73E-05	
ArcZ	4160147	108	+	3234	1708	1694	1923	1.14	8.20E-05	
STnc130	1161203	135	-	2	1	1	1	1.00	1.66E-04	
STnc560	1939628	214	+	132	58	35	33	0.94	2.27E-04	
sraL	5161197	141	-	627	265	252	228	0.90	2.81E-04	
RydB	2439675	61	-	280	102	260	203	0.78	5.76E-04	
RtT_4	1824474	131	-	30	10	13	9	0.69	9.91E-04	
sroC	767984	163	-	3945	1269	1369	946	0.69	9.99E-04	
CRISPR-DR4_2	1058550	28	+	3	1	6	4	0.67	1.16E-03	
STnc100_2	1267542	167	+	3718	1129	1259	822	0.65	1.27E-03	
sok_15/sokX	3674872	152	-	93	28	35	22	0.63	1.49E-03	tcaggtata
STnc100_4	1641323	191	+	4486	1215	1329	773	0.58	2.02E-03	positive
GcvB	3732394	206	+	13532	3307	3716	1952	0.53	2.96E-03	negative/tgagccgga
Spot_42/spf	4914606	119	+	323	77	154	79	0.51	3.22E-03	gtagggtac
STnc450	5326800	58	-	20	5	20	10	0.50	3.52E-03	
CRISPR-DR4_1	1058490	28	+	4	1	8	4	0.50	3.52E-03	
STAXI_4	1482887	131	+	4	1	2	1	0.50	3.52E-03	
RybB	1014999	79	-	1953	439	1398	676	0.48	3.95E-03	gcagggcat
sroB	572997	84	+	704	151	474	219	0.46	4.59E-03	
P26	5058572	62	+	261	52	238	102	0.43	5.83E-03	
sok_14	2777459	175	-	1539	298	497	207	0.42	6.35E-03	tgaggccca
sroH	5068058	161	-	606	114	213	86	0.40	6.97E-03	
DicF_2	1881271	52	-	5	1	5	2	0.40	7.16E-03	
rdlD_3	1807675	60	+	58	10	55	20	0.36	9.36E-03	
OrzO-P_1	2953705	74	+	7227	1195	5524	1963	0.36	9.96E-03	
sok_10	1888482	175	+	3663	598	1184	415	0.35	1.03E-02	tgaggctca
ryfA	3444344	305	+	16	3	3	1	0.33	1.18E-02	
IS061	2172064	180	-	10	1	3	1	0.33	1.18E-02	
rdlD_4	4509509	66	+	78	11	67	20	0.30	1.49E-02	
rdlD_2	1807146	60	+	59	8	56	16	0.30	0.02	
sok_7	1480784	158	+	2602	366	932	282	0.29	0.02	
RyeB	2600241	100	-	2380	314	1346	382	0.28	0.02	
QUAD_1	2898598	149	+	358	47	136	38	0.28	0.02	
MicF	3117339	94	+	1059	132	637	171	0.27	0.02	
STnc100_6	1893978	190	+	6373	703	1897	450	0.24	0.03	
OxyS	5033797	110	-	106	11	55	12	0.22	0.03	
arrS	4467416	69	-	266	22	201	36	0.18	0.04	
istR	4712705	130	-	99	8	43	7	0.16	0.05	
SraB	1590770	169	+	511	38	171	27	0.16	0.05	
QUAD_6	4001742	150	-	771	54	291	44	0.15	0.06	
DsrA	2725072	87	-	82	6	53	8	0.15	0.06	
StyR-44_7	5087479	109	+	1784	125	926	139	0.15	0.06	
QUAD_5	3861645	151	+	1621	113	607	91	0.15	0.06	
StyR-44_5	4902290	109	+	1846	127	958	142	0.15	0.06	
QUAD_4	3861252	151	+	2395	153	897	123	0.14	0.07	
StyR-44_4	4806012	109	+	1761	112	914	125	0.14	0.07	
StyR-44_1	228975	109	+	1908	111	990	124	0.13	0.08	
STnc240	2830003	75	-	112	6	84	10	0.12	0.08	
Bacteria_small_SRP /ffs	542524	97	+	230378	12741	134343	15969	0.12	0.08	positive
STnc100_9	2773346	167	-	3475	184	1177	134	0.11	0.09	
GlmZ_SraJ_2	4848834	207	+	7351	364	2009	214	0.11	0.10	positive
SraC_RyeA	2600138	145	+	2011	91	784	76	0.10	0.12	
GlmY_tke1_2	4848836	149	+	7310	323	2775	264	0.10	0.12	
StyR-44_6	5046470	109	+	4004	161	2078	180	0.09	0.14	
STnc100_8	2314989	167	-	706	23	239	17	0.07	0.19	
RtT_1	867059	143	+	3357	102	1328	87	0.07	0.21	
C4_2	2673794	88	+	108363	3042	69654	4203	0.06	0.23	
sok_6	1389612	175	-	934	22	302	15	0.05	0.29	
STnc100_7	2145571	190	-	327	7	97	4	0.04	0.35	
CsrB	3714213	360	-	43044	748	6763	253	0.04	0.38	
CsrC	4915753	254	+	25764	425	5738	203	0.04	0.40	
RydC	2079463	64	+	1636	27	1446	51	0.04	0.40	
RNaseP_bact_a /rnpB	4077043	377	-	39359	640	5905	206	0.03	0.40	
GlmZ_SraJ_1	3481543	185	-	7668	122	2345	80	0.03	0.41	
GlmY_tke1_1	3481544	148	-	7634	119	2918	98	0.03	0.42	
6S/ssrS	3860420	184	+	470148	7239	144532	4783	0.03	0.42	
QUAD_3	2899260	144	+	3436	44	1350	37	0.03	0.47	
symR	5467620	77	+	726	6	533	9	0.02	0.60	
sRNA-Xcc1	1392052	89	-	40293	290	25609	396	0.02	0.62	
rdlD_1	1806611	66	+	2090	8	1791	15	0.01	0.76	
StyR-44_3	4229125	109	-	2523	2	1309	2	0.00	0.96	
StyR-44_2	3519339	109	-	2499	1	1297	1	0.00	0.97	
HPnc0260	2421623	163	-	1	0	0	0	N/A	N/A	
rseX	2733408	90	+	4	0	3	0	N/A	N/A	
sok_12	2152486	125	-	13	0	6	0	N/A	N/A	
SraG	4120940	172	+	1	0	0	0	N/A	N/A	
STAXI_1	1087216	64	+	6	0	5	0	N/A	N/A	
STAXI_2	1087280	131	+	2	0	1	0	N/A	N/A	
STAXI_3	1482823	64	+	3	0	3	0	N/A	N/A	
STnc100_11	3553828	189	-	387	0	116	0	N/A	N/A	
STnc410	4777710	158	+	3	0	1	0	N/A	N/A	
tp2	127504	114	-	1	0	0	0	N/A	N/A	
sraA	524870	96	-	0	0	0	0	N/A	N/A	
STnc480	635390	67	+	0	0	0	0	N/A	N/A	
sar	1661162	67	-	0	0	0	0	N/A	N/A	
group-II-D1D4-2	2037712	118	-	0	0	0	0	N/A	N/A	
DicF_3	2159230	56	+	0	0	0	0	N/A	N/A	
C0465	2649880	76	+	0	0	0	0	N/A	N/A	
STnc430	5118969	150	-	0	0	0	0	N/A	N/A	

*; The *P* values give the probability that the RCV of the given RNA is similar to / results from the RCV distribution of the tRNAs. Thus, RNAs with high *P* values are probably not translated and *vice versa*

Annotated ncRNAs which are not independent of translation (e.g. leader peptides or ribosomal RNAs, etc.) are not shown (see text). The genome position (start) of each ncRNA is indicated, the ncRNAs are sorted according to their RCV. Transcripts examined via Northern blots are indicated and putative Shine-Dalgarno sequences are shown. An overview of all data for the 115 known ncRNAs is found in Additional file 8: Table S6

**Fig. 2:**
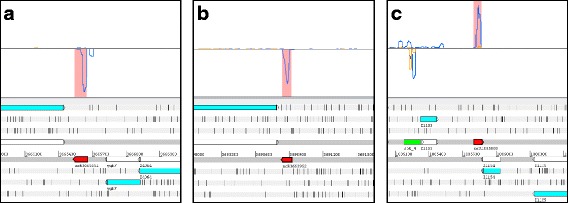
Three examples of novel ncRNAs detected using transcriptome and translatome analysis. A genomic area is visualized in Artemis 15.0.0 [43]. In the lower part of the panels, the genome (shown as *grey lines*) is visualized in a six-frame translation mode. Numbers given between the grey lines indicate the genome coordinates. On top of the forward strand are three reading frames and on the reverse DNA strand are three further reading frames. Each reading frame represented is visible by the indicated stop codons (*vertical black bars*). Annotated genes are shown in their respective reading frame (*turquoise arrows*) and also on the DNA strand itself (*white arrows*). The gene name is written below each arrow. Any protein-coding ORF must be at least located between two *black bars*, with the downstream stop codon being the translational stop. In the upper part of the panels, the DNA is indicated by a *thin black line* and the sequencing reads matching to the forward or reverse strand are shown above or below this line. The sequencing reads from the footprint (*yellow line*) and transcriptome (*blue line*) sequencing are shown as coverage plot, respectively. The pink shaded area in the coverage plot corresponds to the novel ncRNAs, which are drawn in by red arrows. Novel ncRNAs were identified by their very low RCV, thus, hardly any footprint reads (in *yellow*) but a number of transcriptome reads (in *blue*; see Table 2). Known ncRNAs are indicated on the DNA by a bright green arrow. Since ncRNAs supposedly do not contain a protein-coding ORF, these genes are only shown on the DNA. **a** ncR3665651. **b** ncR3690952. **c** ncR1085800

Using the RCV limits mentioned in the methods section (i.e., RCV <0.197), 150 putative ncRNAs were discovered of which three examples are shown in Fig. 2. All novel ncRNA candidates are listed in Table 2, including the read counts, RPKM values and RCV values for each transcript. The putative novel ncRNAs range between 27 and 268 nt with an average size of 77 nt. One (ncR3609372) had a match in the Rfam database [56] as being a tRNA. We analyzed these transcripts to see whether they contained a potentially protein coding ORF. Of the 150 identified transcripts, 44 do not contain any ORF at all and only a minority of 6 candidates contains a putative ORF coding for more than 30 amino acids, indicating that most transcripts identified are truly non-coding. This agrees with the fact that all RCVs are below the threshold for translation. The RPKM-transcriptome values of the novel ncRNA transcripts range between 8 and 8857, the average being 198 (Table 2).

**Table 2:** Novel non-coding RNA (ncRNA) candidates (150 in total) based on transcriptome sequencing and ribosomal profiling. ncRNAs are identified by their start position on the genome given in the name (abbreviated as ncR#)

Name	Length	Max. ORF (nt)	Strand	Number of transcriptome reads	Number of footprint reads	RPKM transcriptome	RPKM footprints	RCV	Northern Blot	CopraRNA	CopraRNA term
ncR1085800	72	-	+	11274	532	8857	898	0.1	positive		
ncR1481381	99	9	+	11208	516	6404	634	0.1	positive		
ncR3690952	145	54	-	4898	26	1911	22	0.01	negative	1.18	ecc00620:Pyruvate metabolism
ncR1636218	78	27	-	1034	22	750	34	0.05	negative	1.31^a^	GO:0042364~water-soluble vitamin biosynthetic process
ncR3545929	41	-	+	542	2	748	6	0.01			
ncR3665651	109	45	-	1442	2	748	2	0	negative	1.24	GO:0045426~quinone cofactor biosynthetic process
ncR3860554	73	24	-	637	3	494	5	0.01		0.98	2Fe-2S
ncR1088953	37	-	+	295	4	451	13	0.03			
ncR3066135	48	-	+	354	0	417	0	0			
ncR1484560	42	-	+	294	0	396	0	0			
ncR5355946	47	21	-	246	4	296	10	0.04			
ncR16920	36	-	-	164	4	258	14	0.05			
ncR165975	49	9	+	216	4	249	10	0.04	negative		
ncR5223290	66	-	+	265	3	227	6	0.02			
ncR1641710	114	105	-	430	28	213	30	0.14			
ncR1765944	114	54	+	406	33	201	35	0.18		5.48^a^	membrane
ncR2358348	45	-	-	151	1	190	3	0.01			
ncR1888606	46	18	-	147	4	181	11	0.06			
ncR2530362	64	6	-	196	0	173	0	0			
ncR5133665	29	-	-	86	0	168	0	0			
ncR2638864	38	-	-	100	2	149	6	0.04			
ncR326492	42	-	+	110	9	148	26	0.18			
ncR622277	95	63	+	248	1	148	1	0.01	negative		
ncR2549762	59	45	-	150	2	144	4	0.03			
ncR2287	90	45	-	218	8	137	11	0.08			
ncR1019437	57	-	-	130	2	129	4	0.03			
ncR1694864	51	18	+	116	1	129	2	0.02			
ncR1864748	174	105	-	370	32	120	22	0.19		4.00^a^	cell membrane
ncR3526958	96	45	-	193	9	114	11	0.1		2.92^a^	ecd00190:Oxidative phosphorylation
ncR867065	45	21	-	76	1	96	3	0.03			
ncR1079732	27	-	-	41	2	86	9	0.11			
ncR3020266	116	57	+	168	9	82	9	0.12			
ncR1328373	40	21	-	55	1	78	3	0.04			
ncR3094200	36	9	+	47	3	74	10	0.14			
ncR774709	58	39	+	74	1	72	2	0.03			
ncR3725111	44	-	-	52	1	67	3	0.04			
ncR451977	43	21	-	50	3	66	8	0.13			
ncR4881271	105	75	+	123	7	66	8	0.12		1.70^a^	GO:0022900~electron transport chain
ncR4922734	44	27	+	49	2	63	6	0.09			
ncR1748457	38	9	-	40	0	60	0	0			
ncR4393950	74	-	-	78	5	60	8	0.14			
ncR5324582	92	24	+	94	4	58	5	0.09			
ncR1847082	38	-	+	38	1	57	3	0.06			
ncR2820623	107	66	-	105	9	56	10	0.18		1.89^a^	lipoprotein
ncR1114186	94	60	-	91	3	55	4	0.07			
ncR3583650	35	-	-	34	1	55	3	0.06			
ncR1509794	96	60	-	91	5	54	6	0.12			
ncR4391372	28	-	-	26	2	53	9	0.17			
ncR4546182	36	12	-	34	2	53	7	0.13			
ncR612919	36	-	+	32	0	50	0	0			
ncR426804	47	-	+	41	3	49	8	0.16			
ncR3164662	66	9	+	56	3	48	6	0.12			
ncR2585184	44	-	+	36	1	46	3	0.06			
ncR2930972	38	6	-	30	2	45	6	0.14			
ncR3527530	119	51	-	95	4	45	4	0.09		1.97^a^	GO:0046395~carboxylic acid catabolic process
ncR4520884	50	24	+	40	3	45	7	0.16			
ncR2498369	53	33	-	41	1	44	2	0.05			
ncR4161484	42	39	+	33	1	44	3	0.07			
ncR2699447	35	18	+	26	1	42	3	0.08			
ncR5210782	28	18	+	21	0	42	0	0			
ncR205409	74	45	-	53	0	41	0	0		1.02	IPR014021:Helicase, superfamily 1 and 2, ATP-binding
ncR1868696	103	30	-	72	6	40	7	0.18			
ncR3915561	37	-	-	26	2	40	7	0.17			
ncR1462015	40	21	-	27	0	38	0	0			
ncR1475353	73	-	-	48	4	37	7	0.18			
ncR397399	68	-	-	44	0	37	0	0			
ncR4645569	57	15	-	36	0	36	0	0			
ncR1239030	54	-	+	33	1	35	2	0.07			
ncR1645154	42	-	-	26	1	35	3	0.08			
ncR3553461	57	33	+	35	1	35	2	0.06			
ncR4853400	65	15	+	40	3	35	6	0.16			
ncR1143400	43	18	+	25	2	33	6	0.17			
ncR2693045	49	6	-	29	2	33	5	0.15			
ncR3735643	69	45	+	40	2	33	4	0.11			
ncR3991822	44	9	-	26	0	33	0	0			
ncR4714439	67	6	-	39	2	33	4	0.11			
ncR1960332	35	-	+	20	1	32	3	0.11		1.96^a^	GO:0034660~ncRNA metabolic process
ncR2885483	44	-	-	25	1	32	3	0.09			
ncR4463425	63	12	-	36	1	32	2	0.06			
ncR501481	70	21	+	39	0	32	0	0			
ncR963596	50	9	-	28	1	32	2	0.08			
ncR1152534	90	51	+	50	4	31	5	0.17		0.84	ecq00052:Galactose metabolism
ncR2602372	62	12	-	34	2	31	4	0.13			
ncR2062548	38	-	+	20	0	30	0	0			
ncR4770438	40	12	+	21	1	30	3	0.1			
ncR865067	103	51	-	54	1	30	1	0.04			
ncR11537	73	48	+	37	2	29	3	0.12			
ncR3040352	77	18	-	40	3	29	5	0.16			
ncR4249267	64	9	+	33	2	29	4	0.13			
ncR1592436	48	9	-	24	1	28	3	0.09			
ncR3583545	40	-	-	20	1	28	3	0.11			
ncR725615	61	-	+	30	2	28	4	0.14			
ncR1066434	66	21	-	32	1	27	2	0.07			
ncR4163613	125	45	+	59	5	27	5	0.18		1.29	antibiotic resistance
ncR15950	44	18	+	20	0	26	0	0			
ncR2452385	45	18	-	21	0	26	0	0			
ncR2841773	79	18	+	37	0	26	0	0			
ncR3320428	75	51	+	34	1	26	2	0.06			
ncR2903620	140	33	-	61	3	25	3	0.11			
ncR3042903	164	57	-	69	3	24	2	0.09			
ncR543583	67	51	+	29	0	24	0	0			
ncR1585703	88	48	+	36	2	23	3	0.12			
ncR1999946	51	15	+	21	0	23	0	0			
ncR5077759	50	6	-	20	1	23	2	0.11			
ncR1640190	56	-	-	22	2	22	4	0.2			
ncR1752346	64	-	+	25	0	22	0	0			
ncR3074598	54	36	-	21	0	22	0	0			
ncR3320341	85	18	+	33	0	22	0	0			
ncR3330508	57	-	-	22	0	22	0	0			
ncR4096595	60	-	-	23	2	22	4	0.19			
ncR4137844	268	195	-	102	7	22	3	0.15			
ncR4414172	53	-	-	21	0	22	0	0			
ncR492696	51	-	-	20	0	22	0	0			
ncR1215540	74	21	+	28	2	21	3	0.15			
ncR2254917	76	-	+	28	2	21	3	0.15			
ncR2902855	104	-	-	39	3	21	4	0.17			
ncR752395	172	66	+	64	2	21	1	0.07			
ncR283226	71	27	+	25	0	20	0	0			
ncR1049002	77	48	-	26	1	19	2	0.08		1.84^a^	GO:0050890~cognition
ncR1216838	100	12	-	33	1	19	1	0.07			
ncR4829752	78	-	-	26	1	19	2	0.08			
ncR1483108	77	63	+	23	1	17	2	0.09			
ncR155024	78	18	+	23	0	17	0	0			
ncR4156147	78	-	-	23	1	17	2	0.09		1.75^a^	GO:0032196~transposition
ncR4788281	97	78	-	30	0	17	0	0		0.99	topological domain:Periplasmic
ncR1854285	91	48	-	25	1	16	1	0.09			
ncR1942672	85	-	+	24	1	16	1	0.09		2.09^a^	GO:0015031~protein transport
ncR2614043	86	57	-	25	0	16	0	0			
ncR4741832	75	30	-	21	0	16	0	0			
ncR484751	94	51	-	27	2	16	3	0.16			
ncR5283975	98	84	-	28	0	16	0	0		2.14^a^	GO:0009386~translational attenuation
ncR1314229	101	48	+	26	2	15	2	0.17			
ncR1893573	187	87	-	48	4	15	3	0.18			
ncR3609575	74	-	+	20	0	15	0	0		1.72^a^	eum00660:C5-Branched dibasic acid metabolism
ncR3724967	105	42	-	28	0	15	0	0			
ncR4245110	118	48	+	30	1	14	1	0.07			
ncR866957	79	42	-	20	0	14	0	0			
ncR336953	96	27	+	22	2	13	3	0.2			
ncR3997954	104	81	+	23	2	13	2	0.19			
ncR411208	99	6	-	22	2	13	2	0.2		2.64^a^	GO:0015980~energy derivation by oxidation of organic compounds
ncR1736783	100	57	-	22	1	12	1	0.1			
ncR745748	107	54	+	23	1	12	1	0.09			
ncR3609372	201	-	+	38	1	11	1	0.06			Rfam match: tRNA RF00005^b^
ncR3890479	104	51	-	20	1	11	1	0.11			
ncR1087411	224	135	+	41	2	10	1	0.11			
ncR3874837	127	48	-	22	2	10	2	0.2			
ncR4187491	122	9	-	22	0	10	0	0			
ncR769665	198	102	+	34	2	10	1	0.13			
ncR5157894	218	114	+	33	3	9	2	0.2			
ncR196880	135	81	-	20	1	8	1	0.11		1.23	ecc00330:Arginine and proline metabolism

^a^DAVID enrich. score; signif. ≥ 1.3. ^b^tRNA

The longest potential ORF is indicated for each ncRNA. RPKM values of transcriptome and translatome are shown, as well as the ribosome coverage value (RCV). A transcript is considered non-coding if it has at least 20 reads in the transcriptome data and the RCV is below 0.197 (α = 0.01). Transcripts examined via Northern blots are indicated and CopraRNA functional enrichments are shown

### Presence of novel ncRNAs in *E. coli* K12

In *E. coli* O157:H7 EDL933, 329 ncRNAs have been annotated [2], but various bioinformatic studies suggest the existence of up to 1000 ncRNAs in *E. coli* (e.g. [8–11]) and probably in other bacteria as well (e.g. [19, 79]). Our current study presents even under a single growth condition 150 new ncRNA candidates. For comparison, we determined the presence of corresponding regions in the *E. coli* K12 strain MG1655. We found 102 of 150 novel ncRNAs regions present in MG1655. Next, we searched data of prokaryotes having both, transcriptome and translatome data of the same experiment. Only a single study was published by the Weissman group of MG1655 grown in MOPS glucose medium [80]. In addition, the ArrayExpress database contains a further dataset of MG1655 grown in LB (E-MTAB-2903). In MOPS medium with glucose at OD 0.3 and in LB medium at an OD of about 0.5, 43 and 66 of the 102 putative ncRNAs were found to be transcribed in MG1655, respectively. Combining both datasets confirmed transcription (without translation) of 74 of the 102 ncRNAs under either condition in *E. coli* MG1655 (Additional file 7: Table S5).

### Detection of ncRNAs by Northern blots

**Fig. 3:**
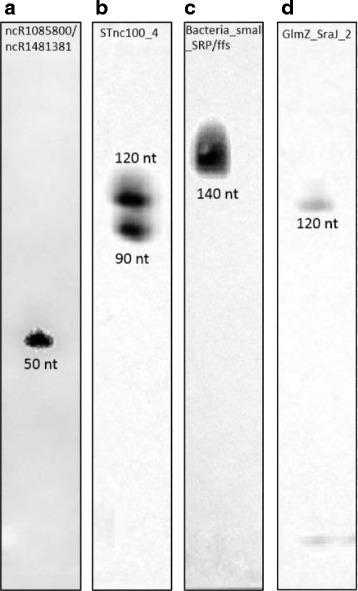
Detection of novel and annotated ncRNAs by Northern blots. Since ncRNAs do not have defined ends like, e.g., ORFs which have start and stop codons, their actual length may differ somewhat from the expected length (compare to Table 1). The contrast of the bands has been adjusted by gamma correction using digital image processing for better visibility. **a** ncR1085800 and ncR1481381. Both ncRNAs are indistinguishable by their sequence. **b** STnc100_4. **c** Bacteria_small SRP/ffs. **d** GlmZ_SraJ_2

To verify the existence of at least some annotated ncRNAs, Northern blot analysis was conducted for five of the annotated ncRNAs of different length and strength. Three were verified, namely *ffs*, *sraJ*, and STnc100_4 (Table 1 and Fig. 3). We then chose seven exemplary novel ncRNAs to be confirmed using Northern blots. However, of the novel ncRNAs only the two transcripts with the highest RPKM in the transcriptome of 8857 and 6404 could be verified as sum signal since they are indistinguishable on the basis of Northern blots (Fig. 3). Obviously, Northern blots have a certain detection limit. Under the conditions applied in this study, any RNA required an RPKM value of about 2000 to be detectable. RNAs transcribed at lower levels were not detected via hybridization. A sufficiently high number of RNA molecules are needed to generate a signal passing the detection threshold, a problem also common to microarrays [81, 82].

### Putative functions and differential expression of the novel ncRNAs

To examine putative functions of the novel ncRNA candidates, we used the sRNA target prediction tool CopraRNA [59, 60]. For 14 of the 150 novel ncRNAs, a significant functional enrichment was found (Table 2). The targets include a diversity of metabolic and regulatory functions within the cell, e.g., synthesis pathways of amino acids and vitamins; but also respiratory functions and oxidation of components etc.

Interestingly, 121 of the novel ncRNAs were found to be expressed (i.e., ≥ 10 RPKM, which is ≥ 100-fold above background) in the data of a former study [17], when grown in eleven different growth conditions for at least one condition. Forty-six novel ncRNAs revealed 4-fold differential expression in at least one other condition when compared to plain LB. Example data are given in Table 3, the full data set can be found in the Additional file 7: Table S5. Combining these findings of CopraRNA predictions (14), Rfam match (1), expression (121), and regulation (46) suggests that at least 126 out of 150 putative ncRNAs are not just a random by-product of pervasive transcriptional activity, but might fulfill specific functions in the cell.

**Table 3:** Expression of exemplary novel ncRNAs under 11 different growth conditions (MM, minimal medium)

Name	Length [nt]	LB plain	MM	LB + nitrite	LB pH9	Radish sprouts	Spinach juice	LB 15°C	LB + antibiotics	Cow dung	LB solid medium	LB pH4
ncR1085800	72	4177	4563	4990	345	25504	3228	1940	655	11683	1410	9815
ncR1114186	94	641	0	416	684	102	29	1382	0	65	246	26
ncR1481381	99	2774	3227	3740	291	19298	2504	1266	411	8632	1108	6997
ncR1483108	77	128	188	223	46	31	79	33	42	0	48	32
ncR1509794	96	628	0	529	628	50	7	1407	0	51	288	43
ncR1641710	114	168	356	662	533	504	183	215	14	21	65	7
ncR1854285	91	153	30	45	10	92	30	34	232	27	8	109
ncR1864748	174	23	26	24	3	28	4	24	262	28	13	19
ncR1868696	103	293	210	353	185	267	59	387	16	106	43	48
ncR1999946	51	34	18	215	43	117	0	251	0	95	0	0
ncR2585184	44	119	41	109	20	27	76	372	0	0	51	0
ncR348122	91	382	30	324	136	355	1440	1152	36	67	172	244
ncR3526958	96	18	28	21	5	212	56	112	0	101	8	69
ncR4137844	268	551	249	558	821	304	283	1113	55	77	265	55
ncR4546182	36	129	75	343	135	0	0	227	0	34	207	0
ncR4853400	65	134	139	369	341	331	341	102	0	75	149	0
ncR612919	36	80	50	38	49	0	224	0	0	34	21	0

The RPKM values for each condition are shown. The experimental setup is described in Landstorfer et al. [17]; data for all novel ncRNAs can be found in Additional file 7: Table S5

### Evidence for translation of annotated ncRNAs

**Fig. 4:**
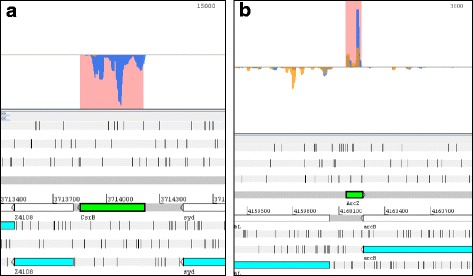
Visualization of ribosomal footprints and transcript reads mapping to annotated ncRNAs as coverage plots. A genomic area is visualized in Artemis 15.0.0 [43]. In the lower part of the panels, the genome (shown as *grey lines*) is visualized in a six-frame translation mode. Numbers given between the grey lines indicate the genome coordinates. On top of the forward strand are three reading frames and on the reverse DNA strand are three further reading frames. Each reading frame represented is visible by the indicated stop codons (*vertical black bars*). Annotated genes are shown in their respective reading frame (*turquoise arrows*) and also on the DNA strand itself (*white arrows*). The gene name is written below each arrow. Any protein-coding ORF must be at least located between two *black bars*, with the downstream stop codon being the translational stop. In the upper part of the panels, the DNA is indicated by a *thin black line* and the sequencing reads matching to the forward or reverse strand are shown above or below this line. The sequencing reads from the footprint (*yellow*) and transcriptome (*blue*) sequencing are shown as filled coverage plots, respectively. The known ncRNAs are indicated on the DNA by a *bright green arrow*. Since ncRNAs supposedly do not contain a protein-coding ORF, these genes are only shown on the DNA. **a** *csrB*: Very few footprint reads are seen for CsrB, indicating that this ncRNA is not translated. **b** *arcZ*: In contrast, ArcZ is covered with many footprints and a number of transcript reads are found. All further examples are shown in Additional file 9: Figure S2

To our own surprise, a significant number of annotated ncRNAs had high RCVs indicating translation, which we examined further. Table 1 shows the known ncRNAs which i) are independent from protein coding genes (i.e., are not leader peptides or riboswitches, etc.), ii) are not ribosomal RNA or iii) do not encode tRNAs. The remaining 115 annotated ncRNAs were categorized according to their RCV (Fig. 1c; Additional file 8: Table S6). As expected for ncRNAs, 52 of these ncRNAs are not translated and have a low RCV (RCV ≤ 0.16). This indicates transcription but no translation. Surprisingly, we identified 52 ncRNAs with an RCV higher than 0.355 (α = 0.01) which we used as lower limit for considering a transcript to be translated (Additional file 9: Figure S2). For both cases, an ncRNA example with low (*csrB*) and high (*arcZ*) RCV is shown in Fig. 4. Eleven ncRNAs fall in an RCV range above the upper limit for untranslated and below the lower limit for translated RNAs and, thus, their translation status (i.e., either untranslated or weakly translated) is difficult to assess. In summary, the ncRNAs were divided into three groups with different ribosome coverage: low RCV similar to untranslated RNAs (52 or 45.5%), such of ambiguous nature (11 or 9%), and those with high RCV similar to translated genes (52 or 45.5%). Clearly, the RCV threshold at which an RNA is considered to be translated depends on the assumed distribution fitted to the tRNA values (see Methods). In any case, different thresholds only alter the region of uncertainty, but do not invalidate our principal finding that quite a number of annotated ncRNAs appear to be associated with ribosomes. Normally, translation is considered the main cause for ribosome binding of an RNA in RIBOseq experiments [83].

We analyzed the potential ORFs of the 52 ncRNAs covered by ribosomes for their annotation status in other organisms using *blastp* [58]. Twenty were found to contain ORFs which achieve blastp-hits to multiple genes annotated in other enterobacteria (e value 10^−3^ or lower), mainly in other *Escherichia coli* strains. From these, 15 are annotated as hypothetical proteins, two belong to toxin-antitoxin systems, one encodes a conserved domain of phage origin and the remaining two are membrane proteins (Additional file 8: Table S6).

### Correlation of translation with Shine-Dalgarno sequences

The presence or absence of a Shine-Dalgarno sequence in proper distance to the start codon can be an indicator for a translational start [66]. A strong Shine-Dalgarno sequence should correspond to a high RCV. On a global scale, i.e. taking average values of all genes with comparable Shine-Dalgarno sequences, such a correlation was found (Additional file 4: File S1). However, predictions are unreliable for single genes. Since several genes exist which either have no Shine-Dalgarno or are completely leaderless [65], a missing Shine-Dalgarno is not necessarily an indication for absent translation. We then searched for the presence of a Shine-Dalgarno sequence for those 20 ncRNAs which have a blastp hit. A start codon in reasonable distance to the start coordinate of the ncRNA was selected (see Methods) and a possible Shine-Dalgarno sequence was determined according to Ma et al. [63], also including weak Shine-Dalgarno sequences. In 11 of 20 cases, a putative Shine-Dalgarno sequence was found (Table 1, Additional file 8: Table S6). The Shine-Dalgarno sequences were also determined as above according to Hyatt et al. [66] (see Additional file 4: File S1), but this method is more stringent and misses some of the weaker sequences (Additional file 8: Table S6). The observation that 11 out of 20 translated ncRNAs with blastP hit (i.e., 55%) have Shine-Dalgarno sequences compares well to about 57% annotated genes possessing such a sequence in *E. coli* K12 [64].

### Why are ncRNAs covered with ribosomes?

Translational profiling showed that 52 annotated ncRNAs have high RCVs. High RCVs may occur due to incomplete digestion of free RNA. Therefore, we had performed two rounds of RNase I digestion and sucrose density gradient centrifugation for ribosomal enrichment, which makes this assumption very unlikely. Most ncRNAs are reported in the Rfam database to bind Hfq and regulate via antisense pairing to their target genes; some ncRNAs are of completely unknown function, and few are involved in toxin-antitoxin interactions. We consider it unlikely that the high numbers of footprints are false-positives in all cases. While the phenomenon of “translated ncRNAs” is highly discussed for eukaryotes [70, 71, 84–89], this observation has, to our knowledge, only rarely been reported for bacteria, i.e. SgrS/SgrT or the “ncRNA” C0343 ([90]; see below, [91]).

In any case, the ribosomal “coverage” of tRNAs (median RCV 0.03), taken as background in this study, is far below the high ribosomal coverage of some ncRNAs. Finally, another explanation for ribosomal coverage of ncRNAs is regulatory functions performed by interaction of the ncRNA with the ribosomes and, thereby, causing accidental carry-over. However, ribosome-interacting ncRNAs are a minority according to Guttman et al. [86].

### RNAs functioning as both ncRNA and mRNA?

A few ncRNAs which are also translated have been suggested to exist in bacteria and are termed coding non-coding RNAs (cncRNAs) [24]. *sgrS*/*sgrT* is the only known example for *E. coli* K12 [90]. In EHEC EDL933, the ATG start codon used by *E. coli* K12 is mutated to ATT. In addition, the Shine-Dalgarno sequence has changed from AAGGGGGT in K12 to AAGGAGGT in EDL933, the very best category S27 of Hyatt et al. [66]. Since a strong SD sequence compensates a weak start codon [63], and *sgrS* has an RCV of 1.55 (Table 1), and ATT is known to be a (very rare) start codon in *E. coli* [92–94], we hypothesize that EHEC synthesizes SgrT using the uncommon start codon ATT. Interestingly, the ORF encoding for SgrT gave a Ka/Ks ratio below 1, i.e. 0.15 with a *P* value of about 0.002. Unfortunately, most ORFs found covered with footprints proved to be too short for any meaningful Ka/Ks analysis (data not shown). Only one other footprint-covered ORF of the ncRNA MicA gave significant results. This ORF had a Ka/Ks ratio of about 0.35 with a *P* value of about 0.018 (Additional file S3: Table S3).

Not all former entities named as ncRNA in the past, however, are cncRNAs. For instance, C0343 had formerly been described as ncRNA, but contains an ORF and yields an RCV of 2.49 in our study (not shown). This validates Washietl et al. [91] who shows that C0343 encodes a short 57-aa protein. Consequently, this entity was possibly falsely labelled as ncRNA and it had been removed from the Rfam database. However, a former study described 72 novel intergenic small protein-coding genes of EHEC [83]. We found six instances in which the locus of a novel protein-coding gene overlaps fully or partly with the locus of one of the ncRNAs (Additional file 8: Table S6), which also hints towards cncRNAs.

In any case, we suggest being cautious in labeling any ribosome covered “ncRNA” of *E. coli* found in this study as cncRNA since further experimental evidence is needed. Based on our current results, we conclude that ribosome covered ncRNAs may represent a mixture of misannotated short mRNAs, ncRNAs with a regulatory function including potential ribosomal binding, and cncRNAs translated indeed. To corroborate this hypothesis about additional cncRNAs and to confirm the existence of novel peptides from so called “non-coding” RNAs as indicated by ribosomal footprints, we tested the footprint-covered ORF of *ryhB* for a phenotype (see below).

### *ryhB* supposedly is a novel cncRNA, encoding the RNA RyhB and a phenotype-causing peptide, RyhP

**Fig. 5:**
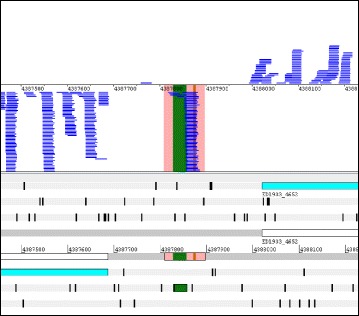
Visualization of individual ribosomal footprints mapping to *rhyB*. The genomic area is visualized in Artemis 15.0.0 [43]. In the lower part of the panels, the genome (shown as *grey lines*) is visualized in a six-frame translation mode. Numbers given between the grey lines indicate the genome coordinates. On top of the forward strand are three reading frames and on the reverse DNA strand are three further reading frames. Each reading frame represented is visible by the indicated stop codons (*vertical black bars*). Annotated genes are shown in their respective reading frame (*turquoise arrows*) and also on the DNA strand itself (*white arrows*). The gene name is written below each arrow. In the upper part of the panels, the DNA is indicated by a thin black line and the footprint reads (*blue*) matching to the forward or reverse strand are shown above or below this line. The shaded areas indicate *ryhB* (*pink*), the coding ORF RyhP (*green*) and a putative weak Shine-Dalgarno sequence (*brown*; ggagaa)

Closer examination of footprint signals for several ncRNAs revealed possible ORFs which encode novel peptides. We chose *ryhB* for further examination, since the encoded RNA-molecule RyhB has a well-known function in iron homeostasis for many bacteria [95–97]. Accordingly, we expected iron-limiting to be the most likely condition in which a phenotype for this novel peptide might be found. Thus, we picked the best matching ORF according to the RIBOseq data, coding for the nona-peptide MAHIASSIT (Fig. 5; start codon ATT) and named it ***ryh*** *B*-encoded **p**eptide, RyhP, in the following. This ORF was introduced on a high-copy arabinose-inducible plasmid in EHEC wild type. In cloning, we omitted all non-coding parts of *ryhB*, to limit any effect the expressed (m)RNA-fragment might have (sequence P1). To even further reduce the possibility that the expressed RNA and not the peptide itself causes the phenotype, we changed all codons of the ORF such that the same peptide is produced, but the underlying RNA sequence differs maximally from the wild type sequence (P2). This strategy prevents the RNA made hybridizing with any natural target RNAs [e.g., 99]. Two negative controls were created, either with the second (T2) or third codon (T3) changed into a stop codon, terminating RyhP translation prematurely. Competitive indices (CI) under RyhB-inducing condition (i.e. low-iron) showed a significant advantage of the strain possessing the RyhP-producing plasmid over those strains containing a plasmid with stop codons in the RyhP-ORF (Table 4).

**Table 4:** Competitive index values (CI) for EHEC strains possessing a wild-type like ORF encoding RyhP (P1 or P2) or an ORF with a premature stop codon (T2 or T3) plusminus their standard deviations (Std)

Wild-type like RyhP-ORF	Terminated RyhP-ORF	CI	±Std
P1	T2	0.79	0.08
P1	T3	0.19	0.08
P2	T3	0.38	0.06

Strains are competitively grown in minimal medium M9 with no iron added for 24 h. The RyhP-encoding ORF was transcriptionally induced with 0.2% arabinose

**Fig. 6:**
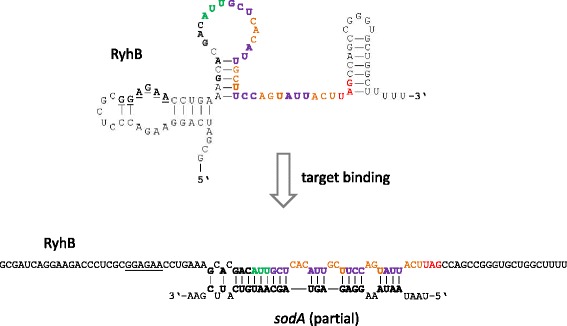
Overview of the secondary structures formed by RyhB for the molecule on its own (*top*) and after binding to a target RNA, like *sodA* (*bottom*). Structures are taken from [99]. Individual bases have been highlighted. *Underlined*, putative Shine-Dalgarno sequence; *green*, start codon; *violet/orange*, individual codons along the frame; *red*, stop codon, bold; bases involved in hybridization to the *sodA*-target

RyhB folds when not bound to its regulated target RNA (Fig. 6) and this, assumedly, makes the coding ORF unavailable for translation. However, ribosomes are able to resolve secondary structures of mRNAs [98]. Furthermore, RyhP has a weak putative Shine-Dalgarno motif (i.e., ggagaa) upstream. Upon binding a target mRNA like *sodA* [99], the RNA structure opens and the Shine-Dalgarno sequence is set free (Fig. 6). If this opening facilitates ribosomal binding for translation initiation of the RyhB RNA, and subsequent progression of ribosomes along the RNA, must remain open.

## Conclusion

In the past, very short proteins or peptides were excluded from annotation and believed to be unlikely. Some short mRNAs could have been labeled as ncRNA solely on this presumption. However, more and more small proteins are being discovered. For instance, a number of small genes have been described for *E. coli* in recent years. These genes were hard to detect because they appear to be membrane proteins and are induced under stress conditions only [100, 101]. In another study, we confirmed the existence of 72 novel and short protein-coding genes in the EHEC genome, some which were verified by proteome data [83]. Similar findings have been made by other groups (see, e.g., [102–104]), and future research could confirm the existence of more of these proteins similar to studies conducted in eukaryotic ribosomal profiling [70, 105–107].

## Additional files

Additional file 1: Table S1. Oligos used in this study to prepare ncRNA-specific probes. (DOC 51 kb)
Additional file 2: Table S2. Bacterial reference genomes used for CopraRNA. (DOCX 15 kb)
Additional file 3: Table S3. Gene pairs for Ka/Ks analysis and results. (XLSX 16 kb)
Additional file 4: File S1. Coverage with ribosomal footprints correlates globally with the conservation of the Shine-Dalgarno sequence. (DOCX 78 kb)
Additional file 5: Figure S1. Correlation of the RPKM translatome between the replicate footprint experiments 1 and 2. (PPTX 142 kb)
Additional file 6: Table S4. Sequencing statistics. The number of mapped reads is listed for the transcriptome and the ribosomal profiling experiments. Additionally, the numbers of reads mapping to rRNA and tRNA genes are shown, as well as the number of remaining reads. (DOCX 14 kb)
Additional file 7: Table S5. Full data set of the 150 novel ncRNAs and their properties in EHEC. Expressed homologs in *E. coli* K12 are indicated, CopraRNA predictions are shown, as well as regulation in eleven diverse growth conditions. (XLSX 81 kb)
Additional file 8: Table S6. Data set of the 115 annotated ncRNAs and their properties, including blastp hits and proposed Shine-Dalgarno sequences. (XLSX 44 kb)
Additional file 9: Figure S2. Overview of 52 known ncRNAs with a translation, i.e. an RCV above the threshold. Panels were drawn using Artemis [43]. (PPTX 5604 kb)

## Abbreviations

aa: Amino acids
blast: Basic local alignment search tool
bp: Base pairs
cncRNA: coding non-coding RNA
dfRNA: dual-functioning RNA
DTT: Dithiothreitol
EHEC: Enterohemorrhagic *E. coli*
LB: Luria-Bertani medium
NEB: New England Biolabs
NGS: Next Generation Sequencing
nt: Nucleotides
OD: Optical density
ORF: Open reading frame
RCV: Ribosome coverage value
RIBOseq: Ribosomal footprinting
r_P_: Pearson correlation
RPKM: Reads per kilobase per million mapped reads
rpm: Revolutions per minute
r_S_: Spearman rank correlation
