# Supplementary material for: Differentiation of ncRNAs from small mRNAs in Escherichia coli O157:H7 EDL933 (EHEC) by combined RNAseq and RIBOseq – ryhB encodes the regulatory RNA RyhB and a peptide, RyhP
Source: BMC Genomics. 2017 Feb 28;18:216. doi: 10.1186/s12864-017-3586-9 (PMC5331693; doi:10.1186/s12864-017-3586-9)
Supplement: Additional file 1: Table S1. — Oligos used in this study to prepare ncRNA-specific probes. (DOC 51 kb) [file 12864_2017_3586_MOESM1_ESM.doc]

| Identifier  **Additional file 1: Table S1.** Oligos used in this study. | Sequence (5’-3’) | Function |
| --- | --- | --- |
| **to prepare ncRNA-specific probes** | | |
| S15683 | GAATTAATACGACTCACTATACGAGTGATATTTTACCATCAACCCGAGG | oligo to prepare sok_1 specific probes |
| S166023 | GAATTAATACGACTCACTATAcagcggttgttttcaaagcaatcgtccgt | oligo to prepare ncR165975 specific probes |
| F542637 | TGTTGGTTCTCCCGCAAC | primer to prepare Bacteria_small_SRP/ffs specific probes |
| R542637 | GAATTAATACGACTCACTATATGCCAGCTACATCCCGGCAC | primer to prepare Bacteria_small_SRP/ffs specific probes |
| S622371 | GAATTAATACGACTCACTATAgttgagagttaccggttttgatatgA | oligo to prepare ncR622277 specific probes |
| F1481479 | gcgatatccggcttattttg | primer to prepare ncR1481381 and ncR1085800 specific probes |
| R1481479 | GAATTAATACGACTCACTATAtggacgcgacatccagcacg | primer to prepare ncR1481381 and ncR1085800 specific probes |
| S1636218 | GAATTAATACGACTCACTATAgagaaaagccccagttaacattacgcgtc | oligo to prepare ncR1636218 specific probes |
| F1641568 | CAATCAGCGAGTAGGTATATG | primer to prepare STnc100_4 specific probes |
| R1641568 | GAATTAATACGACTCACTATACTGGAGAGTGGCGCTATGTG | primer to prepare STnc100_4 specific probes |
| S3665651 | GAATTAATACGACTCACTATAccgaaacacacgatcaatccgaatatgag | oligo to prepare ncR3665651 specific probes |
| F3690808 | GAATTAATACGACTCACTATAGGAACATAAGTTCGACAAC | primer to prepare ncR3690952 specific probes |
| r3690808 | GGATAAACCGAACTAGTTAA | primer to prepare ncR3690952 specific probes |
| F3732595 | TCGGAATGCGTGTTCTGGTG | primer to prepare GcvB specific probes |
| R3732595 | GAATTAATACGACTCACTATACACTATGGACAGACAGGGTA | primer to prepare GcvB specific probes |
| F4849121 | CTTATGTTCGCCTTAGTGCC | primer to prepare GlmZ_SraJ_2 specific probes |
| R4849121 | GAATTAATACGACTCACTATACAGGTCTGTATGACAACAAG | primer to prepare GlmZ_SraJ_2 specific probes |
|  |  |  |
| **to prepare RyhP-encoding frames** | | |
| P1F | CATGGCACATATCGCGAGCTCAATCACGTAA | RyhP-encoding ORF with slight codon changes |
| P1R | CTAGAATGCACTAACTCGAGCGCTATACACG | RyhP-encoding ORF with slight codon changes |
| P2F | CATGGCTCACATTGCCTCCAGTATTACCTGA | RyhP-encoding ORF with maximal codon changes |
| P2R | CTAGAGTCCATTATGACCTCCGTTACACTCG | RyhP-encoding ORF with maximal codon changes |
| T2F | CATGTGACATATCGCGAGCTCAATCACGTAA | premature termination of RyhP at codon 2 |
| T2R | CTAGAATGCACTAACTCGAGCGCTATACAGT | premature termination of RyhP at codon 2 |
| T3F | CATGGCATAGATCGCGAGCTCAATCACGTAA | premature termination of RyhP at codon 3 |
| T3R | CTAGAATGCACTAACTCGAGCGCTAGATACG | premature termination of RyhP at codon 3 |
|  |  |  |
| **to sequence in competitive assays** | | |
| pBAD-C-R | TGATTTAATCTGTATCAGGC |  |
