# Supplementary material for: Differentiation of ncRNAs from small mRNAs in Escherichia coli O157:H7 EDL933 (EHEC) by combined RNAseq and RIBOseq – ryhB encodes the regulatory RNA RyhB and a peptide, RyhP
Source: BMC Genomics. 2017 Feb 28;18:216. doi: 10.1186/s12864-017-3586-9 (PMC5331693; doi:10.1186/s12864-017-3586-9)
Supplement: Additional file 2: Table S2. — Bacterial reference genomes used for CopraRNA. (DOCX 15 kb) [file 12864_2017_3586_MOESM2_ESM.docx]

**Additional file 2: Table S2.** Bacterial reference genomes used for CopraRNA

| **Genbank accession** | **species** | **strain** |
| --- | --- | --- |
| NC_009792 | *Citrobacter koseri* | ATCC BAA-895 |
| NC_013716 | *Citrobacter rodentium* | ICC168 |
| NC_009778 | *Cronobacter sakazakii* | ATCC BAA-894 |
| NC_015663 | *Enterobacter aerogenes* | KCTC 2190 |
| NC_009436 | *Enterobacter* sp. | 638 |
| NC_020063 | Enterobacteriaceae bacterium | FGI 57 |
| NC_008253 | *Escherichia coli* | 536 |
| NC_008563 | *Escherichia coli* | APEC O1 |
| NC_011741 | *Escherichia coli* | IAI1 |
| NC_011750 | *Escherichia coli* | IAI39 |
| NC_002655 | *Escherichia coli* | O157:H7 EDL933 |
| NC_011751 | *Escherichia coli* | UMN026 |
| NC_011740 | *Escherichia fergusonii* | ATCC 35469 |
| NC_000913 | *Escherichichia coli* | K12 |
| NC_009648 | *Klebsiella pneumoniae* subsp. *pneumoniae* | MGH 78578 |
| NC_012917 | *Pectobacterium carotovorum subsp. carotovorum* | PC1 |
| NC_005126 | *Photorhabdus luminescens* subsp. *laumondii* | TTO1 |
| NC_010554 | *Proteus mirabilis* | HI4320 |
| NC_003197 | *Salmonella enterica* subsp. *enterica* | serovar Typhimurium str. LT2 |
| NC_009832 | *Serratia proteamaculans* | 568 |
| NC_007613 | *Shigella boydii* | Sb227 |
| NC_007606 | *Shigella dysenteriae* | Sd197 |
| NC_004337 | *Shigella flexneri* | 2a str. 301 |
| NC_008258 | *Shigella flexneri* | 5 str. 8401 |
| NC_016822 | *Shigella sonnei* | 53G |
| NC_007384 | *Shigella sonnei* | Ss046 |
| NC_007712 | *Sodalis glossinidius* | 'morsitans' |
| NC_008800 | *Yersinia enterocolitica* subsp. *enterocolitica* | 8081 |
| NC_003143 | *Yersinia pestis* | CO92 |
| NC_006155 | *Yersinia pseudotuberculosis* | IP32953 |
