# Supplementary material for: Differentiation of ncRNAs from small mRNAs in Escherichia coli O157:H7 EDL933 (EHEC) by combined RNAseq and RIBOseq – ryhB encodes the regulatory RNA RyhB and a peptide, RyhP
Source: BMC Genomics. 2017 Feb 28;18:216. doi: 10.1186/s12864-017-3586-9 (PMC5331693; doi:10.1186/s12864-017-3586-9)

**Additional file 4: File S1**

**Coverage with ribosomal footprints correlates only after binning to the Shine-Dalgarno sequence**

It could be reasoned that a strong Shine-Dalgarno sequence (i.e., matching the 3’-end of the rRNA and of proper distance to the start codon) correlates to increased translatability, i.e, higher RCVs. A global comparison of the Shine-Dalgarno sequence score for each gene to its RCV showed virtually no correlation (Pearson correlation R = 0.2; data not shown). However, we binned all annotated genes according to the classification of [Hyatt et al. (2010](#_ENREF_35)) used for PRODIGAL, a prokaryotic gene annotation tool (see Table S5 below). For instance, the lowest PRODIGAL bin S0 contains genes with no Shine-Dalgarno sequence detectable, whereas the highest bin S27 contains genes with AGGAGG in the optimal start codon distance of between 5 to 10 bp for *E. coli*. Despite binning, each bin contains genes with an RCV between zero and ≥10 with at first no discernable correlation (Figure S3 below). But surprisingly, both the mean and the median RCV of each bin, correlate quite well with the increasing bin number (R = 0.84 and R = 0.87, respectively). Thus, on a global scale the possession of an optimal Shine-Dalgarno sequence in proper distance to the start codon seem to correlate to the RCV, hence increasing the translatability of the mRNAs. However, post-translational controls seem to override this (weak) global signal for single genes, since all bins, including the low ones, have genes with high RCV and all bins, including the high ones, have genes with an RCV of zero (Figure S3 below).

**Table S5. Shine-Dalgarno RBS Motifs in Prodigal according to** [**Hyatt *et al.* (2010**](#_ENREF_1)**)**

| **Bin #** | **RBS Motif** | **RBS Spacer** |
| --- | --- | --- |
| S0 | None | None |
| S1 | GGA, GAG, AGG | 3-4 bp |
| S2 | GGA, GAG, AGG, AGxAG, GGxGG | 13-15 bp |
| S3 | AGGA, GGAG, GAGG, AGxAGG, AGGxGG | 13-15 bp |
| S4 | AGxAG | 11-12 bp |
| S5 | AGxAG | 3-4 bp |
| S6 | GGA, GAG, AGG | 11-12 bp |
| S7 | GGxGG | 11-12 bp |
| S8 | GGxGG | 3-4 bp |
| S9 | AGxAG | 5-10 bp |
| S10 | AGGAG, GGAGG, AGGAGG | 13-15 bp |
| S11 | AGGA, GGAG, GAGG | 3-4 bp |
| S12 | AGGA, GGAG, GAGG | 11-12 bp |
| S13 | GGA, GAG, AGG | 5-10 bp |
| S14 | GGxGG | 5-10 bp |
| S15 | AGGA | 5-10 bp |
| S16 | GGAG, GAGG | 5-10 bp |
| S17 | AGxAGG, AGGxGG | 11-12 bp |
| S18 | AGxAGG, AGGxGG | 3-4 bp |
| S19 | AGxAGG, AGGxGG | 5-10 bp |
| S20 | AGGAG, GGAGG | 11-12 bp |
| S21 | AGGAG | 3-4 bp |
| S22 | AGGAG | 5-10 bp |
| S23 | GGAGG | 3-4 bp |
| S24 | GGAGG | 5-10 bp |
| S25 | AGGAGG | 11-12 bp |
| S26 | AGGAGG | 3-4 bp |
| S27 | AGGAGG | 5-10 bp |

**Figure S3 (overleaf).** Correlation between the RCVs (Y-axis) of all annotated genes and their category of the Shine-Dalgarno sequence bins (S0 to S27 as indicated, compare to Table S5). Bins with zero members are omitted and bins with less than 100 genes were combined.

**Upper panel**, grey boxes comprise 50% of the genes in each bin centered around the median. Genes ouside the boxes are shown individually (◇). The mean and median are indicated by blue and red diamonds. Each a trendline is shown in the respective color.

**Lower panel**, only the boxes comprising 50% of the genes in each bin centered around the median are shown for convenience. Interestingly, mean and median correlate quite well to the increasing bin number (R = 0.84 and R = 0.87, respectively), but note the small increase of the RCV for those values and the large overlap in RCV values for the bins.


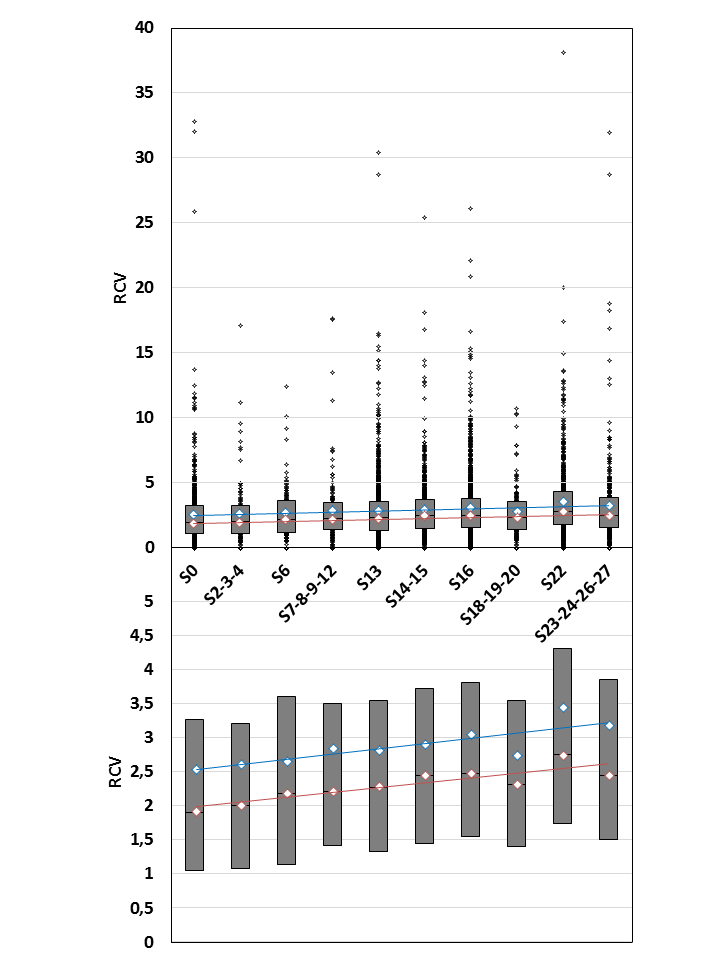

Supplement: Additional file 4: File S1. — Coverage with ribosomal footprints correlates globally with the conservation of the Shine-Dalgarno sequence. (DOCX 78 kb) [file 12864_2017_3586_MOESM4_ESM.docx]
