# Supplementary material for: Differentiation of ncRNAs from small mRNAs in Escherichia coli O157:H7 EDL933 (EHEC) by combined RNAseq and RIBOseq – ryhB encodes the regulatory RNA RyhB and a peptide, RyhP
Source: BMC Genomics. 2017 Feb 28;18:216. doi: 10.1186/s12864-017-3586-9 (PMC5331693; doi:10.1186/s12864-017-3586-9)
Supplement: Additional file 5: Figure S1. — Correlation of the RPKM translatome between the replicate footprint experiments 1 and 2. (PPTX 142 kb) [file 12864_2017_3586_MOESM5_ESM.pptx]

## Slide 1
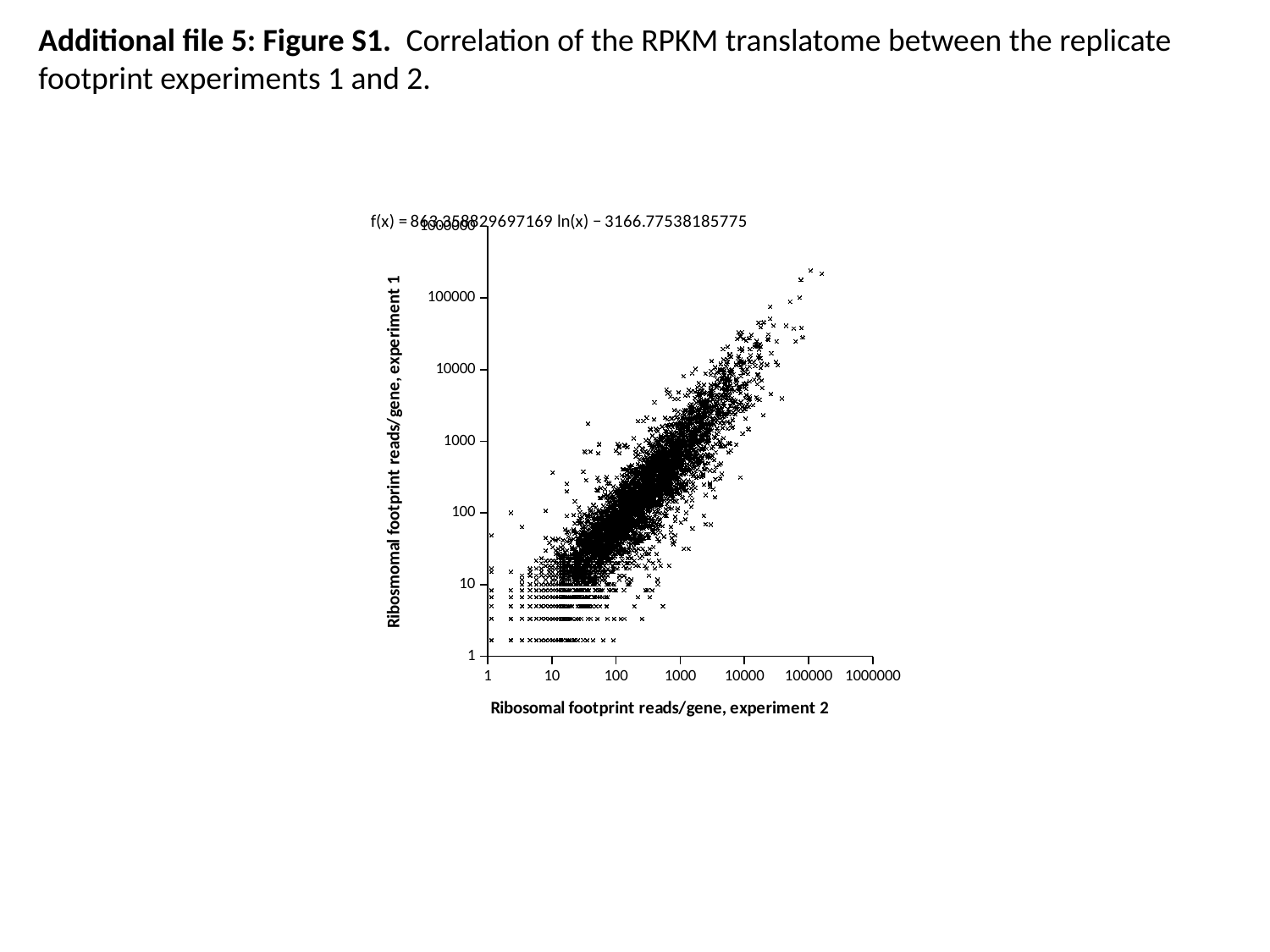

Additional file 5: Figure S1. Correlation of the RPKM translatome between the replicate footprint experiments 1 and 2.
### Chart
| Category | |
|---|---|
