# Supplementary material for: Differentiation of ncRNAs from small mRNAs in Escherichia coli O157:H7 EDL933 (EHEC) by combined RNAseq and RIBOseq – ryhB encodes the regulatory RNA RyhB and a peptide, RyhP
Source: BMC Genomics. 2017 Feb 28;18:216. doi: 10.1186/s12864-017-3586-9 (PMC5331693; doi:10.1186/s12864-017-3586-9)
Supplement: Additional file 6: Table S4. — Sequencing statistics. The number of mapped reads is listed for the transcriptome and the ribosomal profiling experiments. Additionally, the numbers of reads mapping to rRNA and tRNA genes are shown, as well as the number of remaining reads. (DOCX 14 kb) [file 12864_2017_3586_MOESM6_ESM.docx]

**Additional file 6: Table S4.** Sequencing statistics

| **Replicate** | **Mappable reads per genome** | **Reads mapping to tRNA** | **Reads mapping to rRNA** | **Reads mapping to the genome minus rRNA and tRNA** |
| --- | --- | --- | --- | --- |
| Transcriptome repl. 1 | 21,207,616 | 7,959,708 | 8,486,589 | 4,761,319 |
| Transcriptome repl. 2 | 10,808,789 | 1,995,506 | 5,851,016 | 2,962,267 |
| Footprint repl. 1 | 11,170,676 | 26,865 | 6,195,654 | 4,948,157 |
| Footprint repl. 2 | 9,425,184 | 29,656 | 6,174,846 | 3,220,682 |
| Total | 52,612,265 | 10,011,735 | 26,708,105 | 15,892,425 |
