# Supplementary material for: Differentiation of ncRNAs from small mRNAs in Escherichia coli O157:H7 EDL933 (EHEC) by combined RNAseq and RIBOseq – ryhB encodes the regulatory RNA RyhB and a peptide, RyhP
Source: BMC Genomics. 2017 Feb 28;18:216. doi: 10.1186/s12864-017-3586-9 (PMC5331693; doi:10.1186/s12864-017-3586-9)
Supplement: Additional file 9: Figure S2. — Overview of 52 known ncRNAs with a translation, i.e. an RCV above the threshold. Panels were drawn using Artemis [43]. (PPTX 5604 kb) [file 12864_2017_3586_MOESM9_ESM.pptx]

## Slide 1
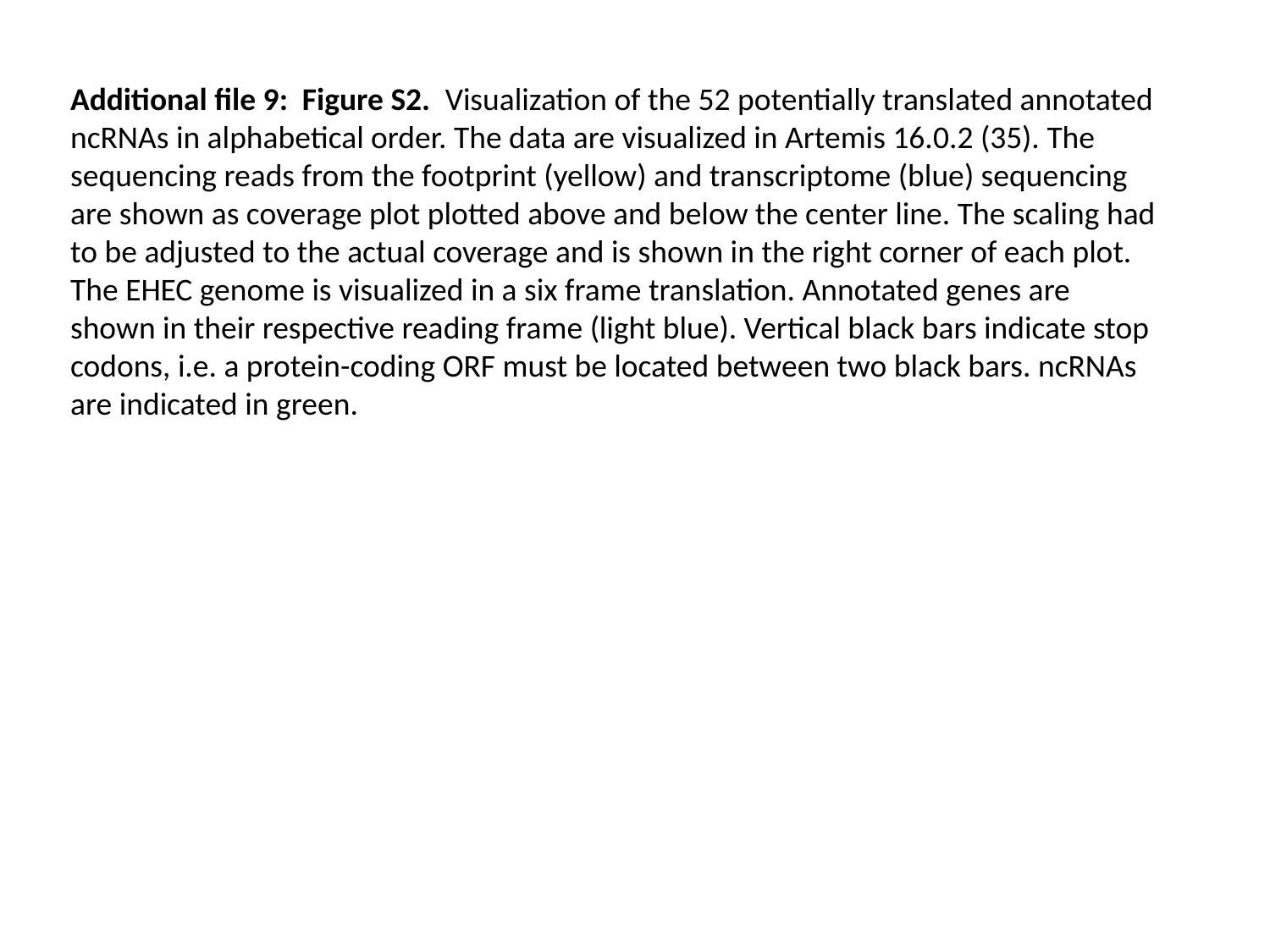

Additional file 9: Figure S2. Visualization of the 52 potentially translated annotated ncRNAs in alphabetical order. The data are visualized in Artemis 16.0.2 (35). The sequencing reads from the footprint (yellow) and transcriptome (blue) sequencing are shown as coverage plot plotted above and below the center line. The scaling had to be adjusted to the actual coverage and is shown in the right corner of each plot. The EHEC genome is visualized in a six frame translation. Annotated genes are shown in their respective reading frame (light blue). Vertical black bars indicate stop codons, i.e. a protein-coding ORF must be located between two black bars. ncRNAs are indicated in green.

## Slide 2
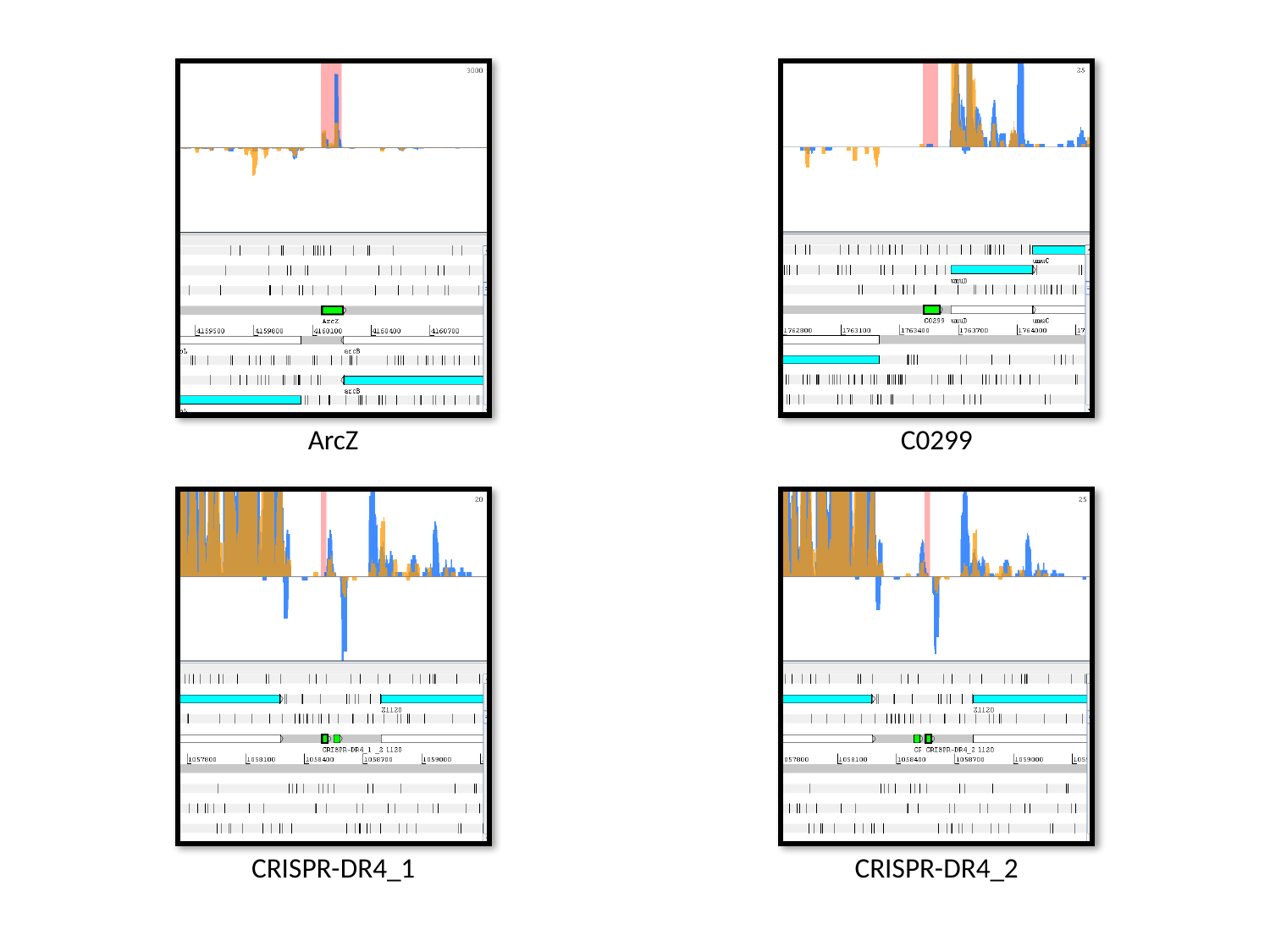

ArcZ
C0299
CRISPR-DR4_1
CRISPR-DR4_2

## Slide 3
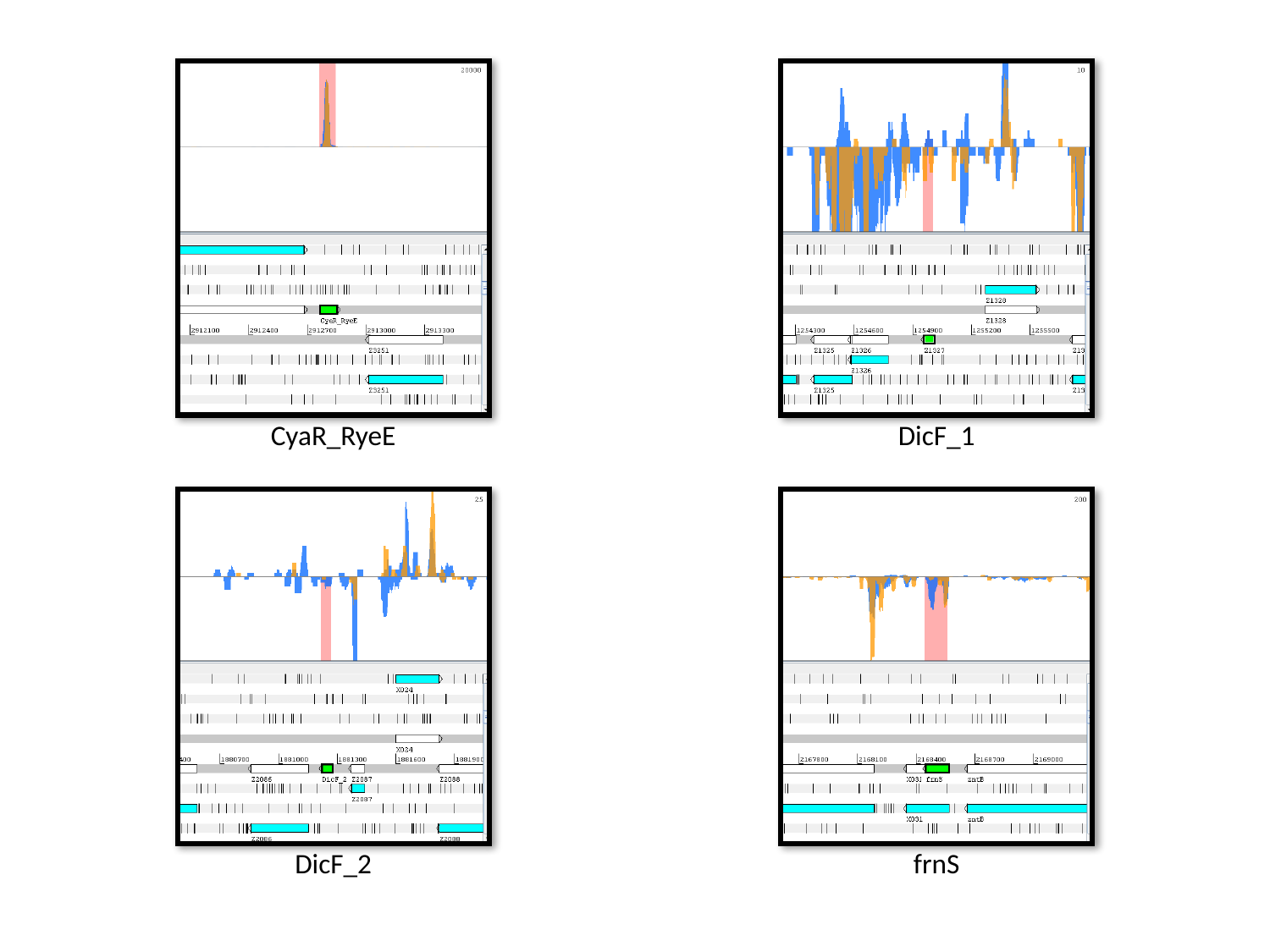

CyaR_RyeE
DicF_1
DicF_2
frnS

## Slide 4
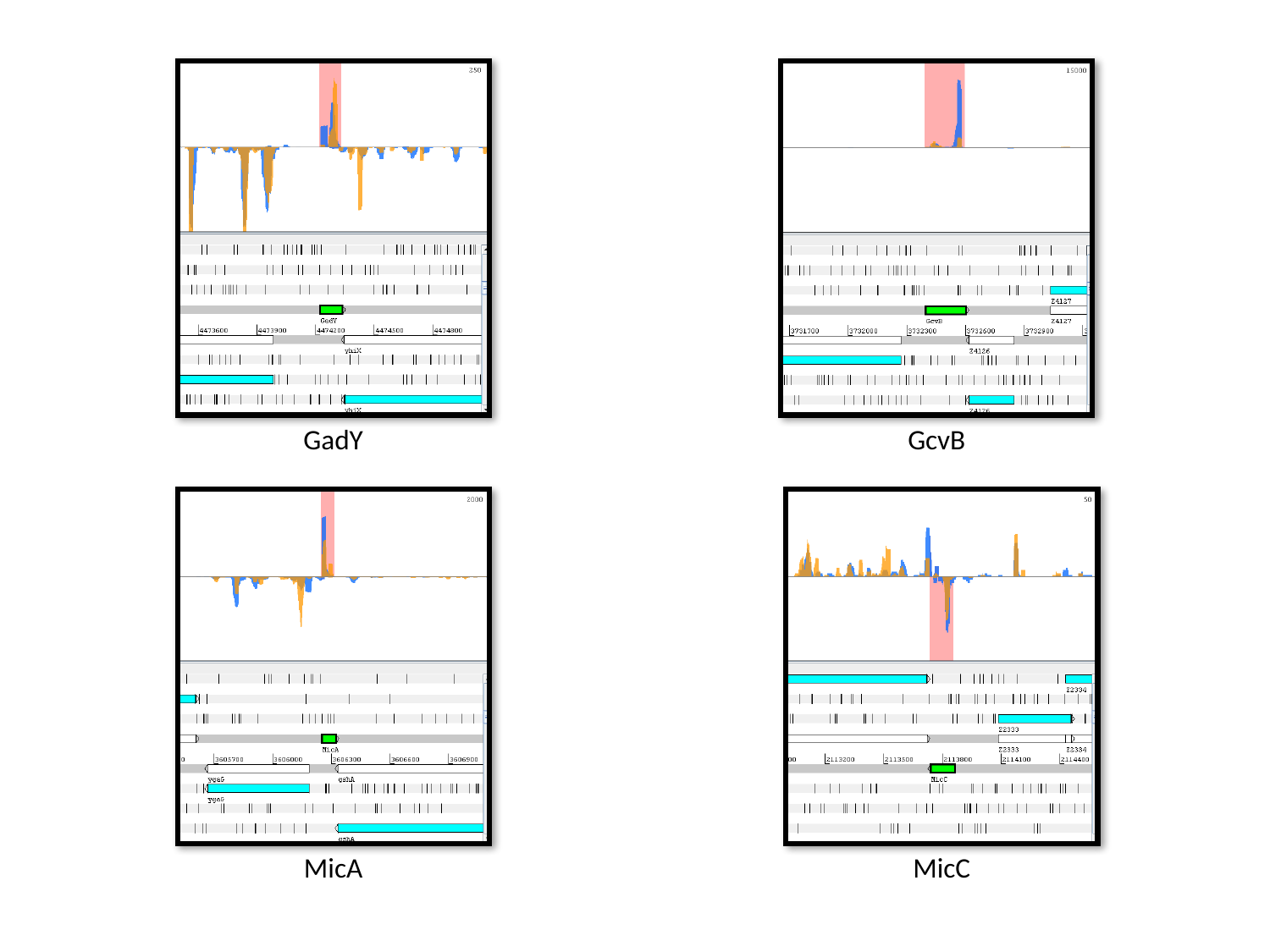

GadY
GcvB
MicA
MicC

## Slide 5
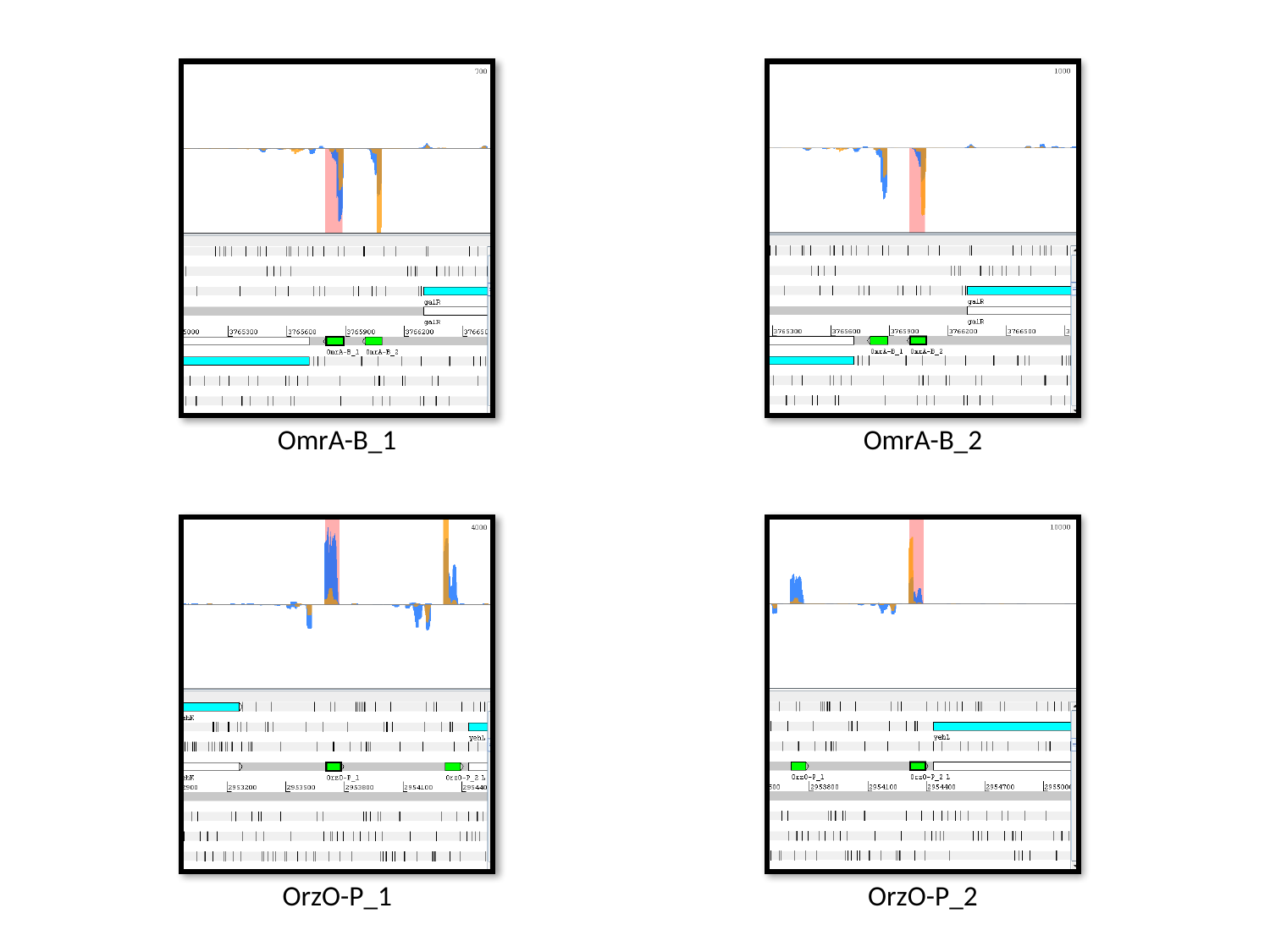

OmrA-B_1
OmrA-B_2
OrzO-P_1
OrzO-P_2

## Slide 6
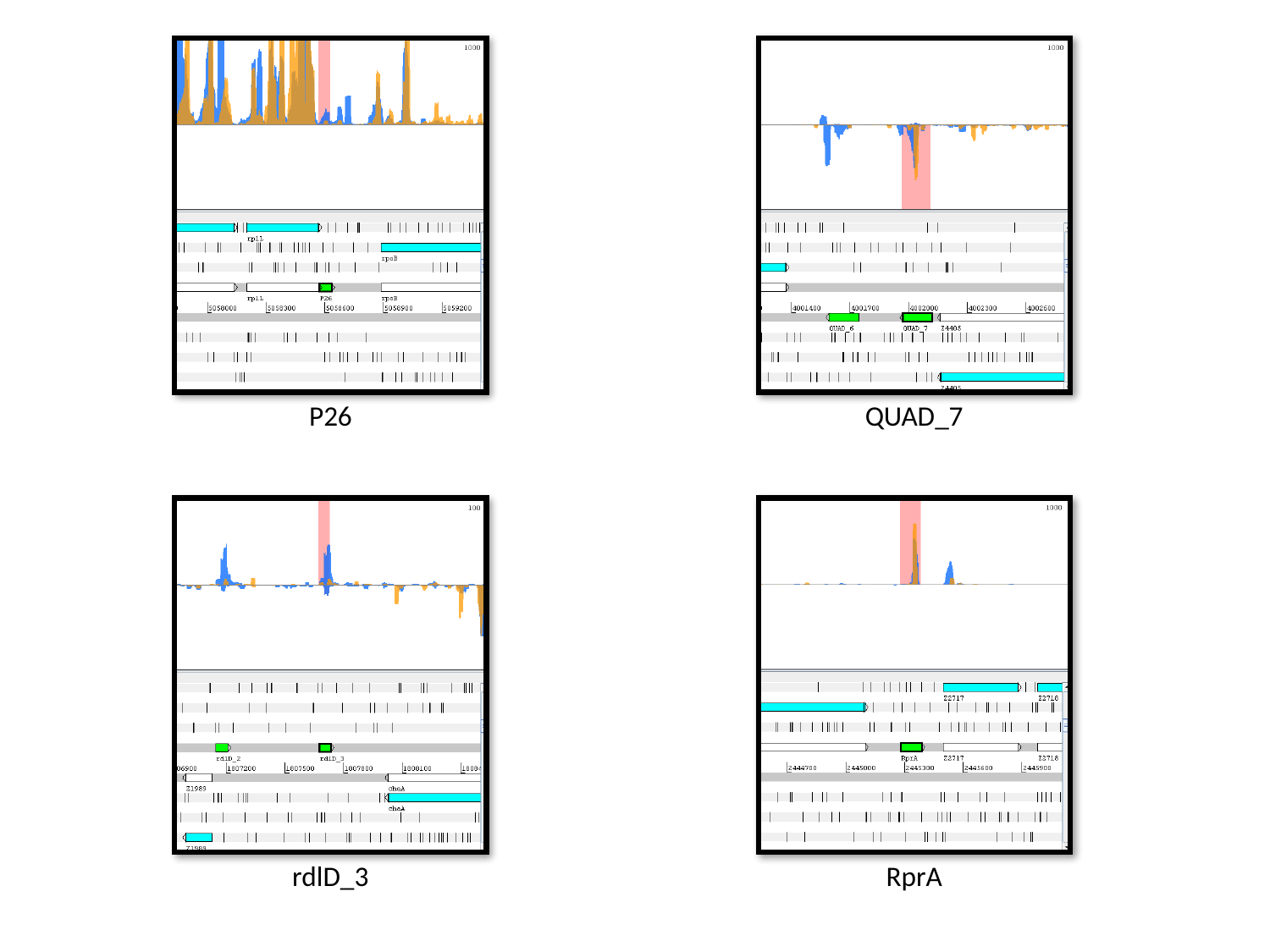

P26
QUAD_7
rdlD_3
RprA

## Slide 7
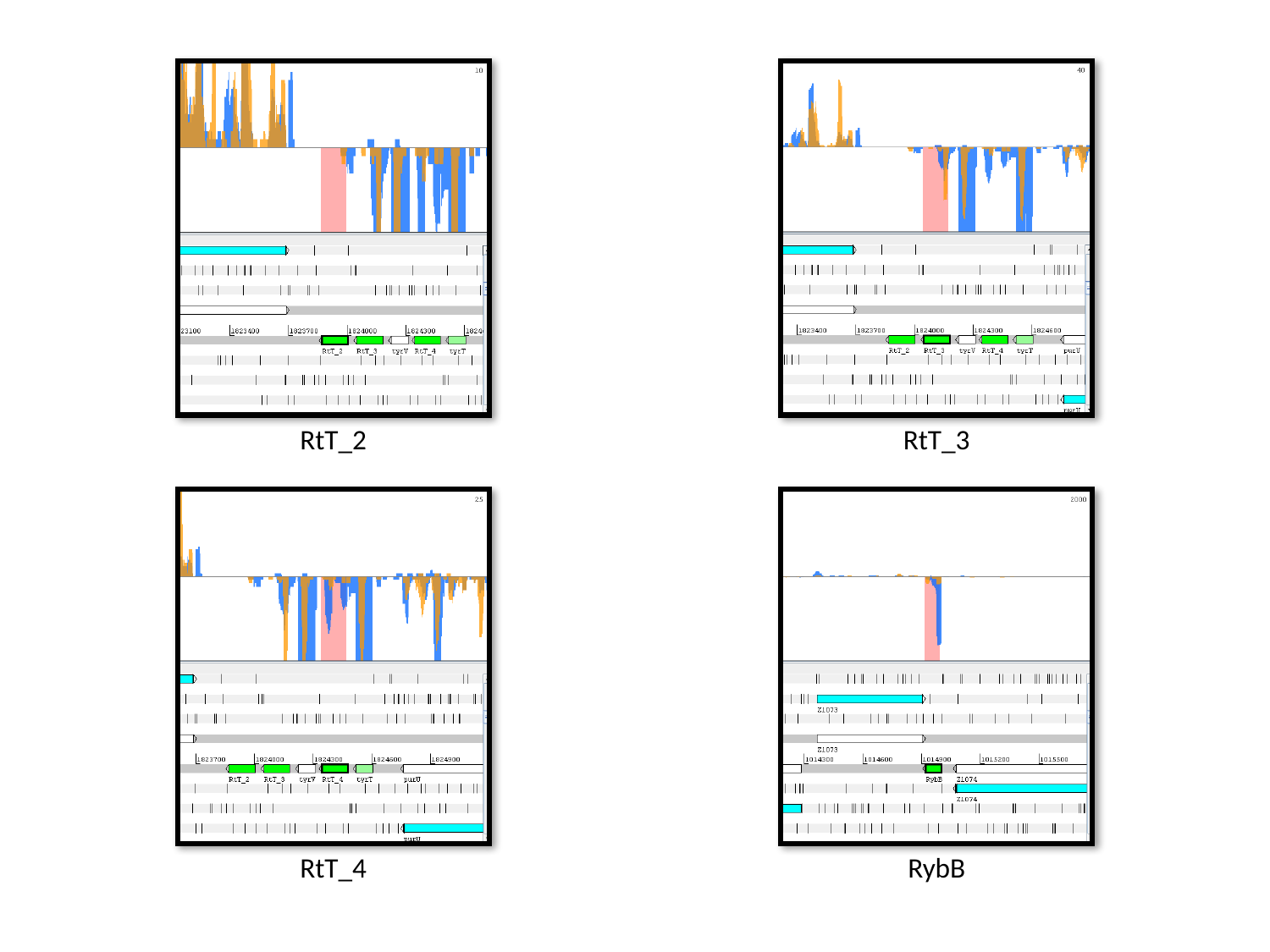

RtT_2
RtT_3
RtT_4
RybB

## Slide 8
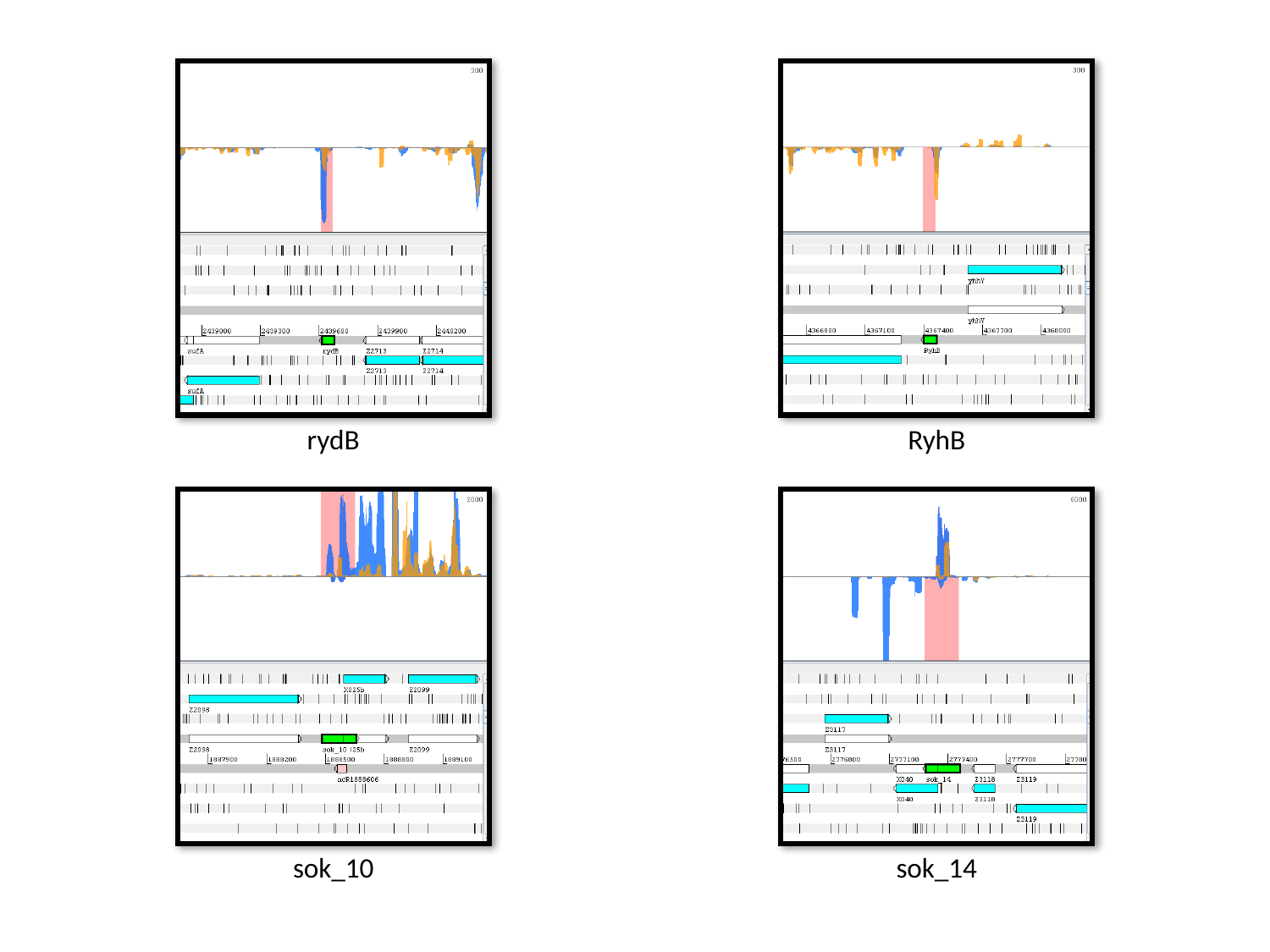

rydB
RyhB
sok_10
sok_14

## Slide 9
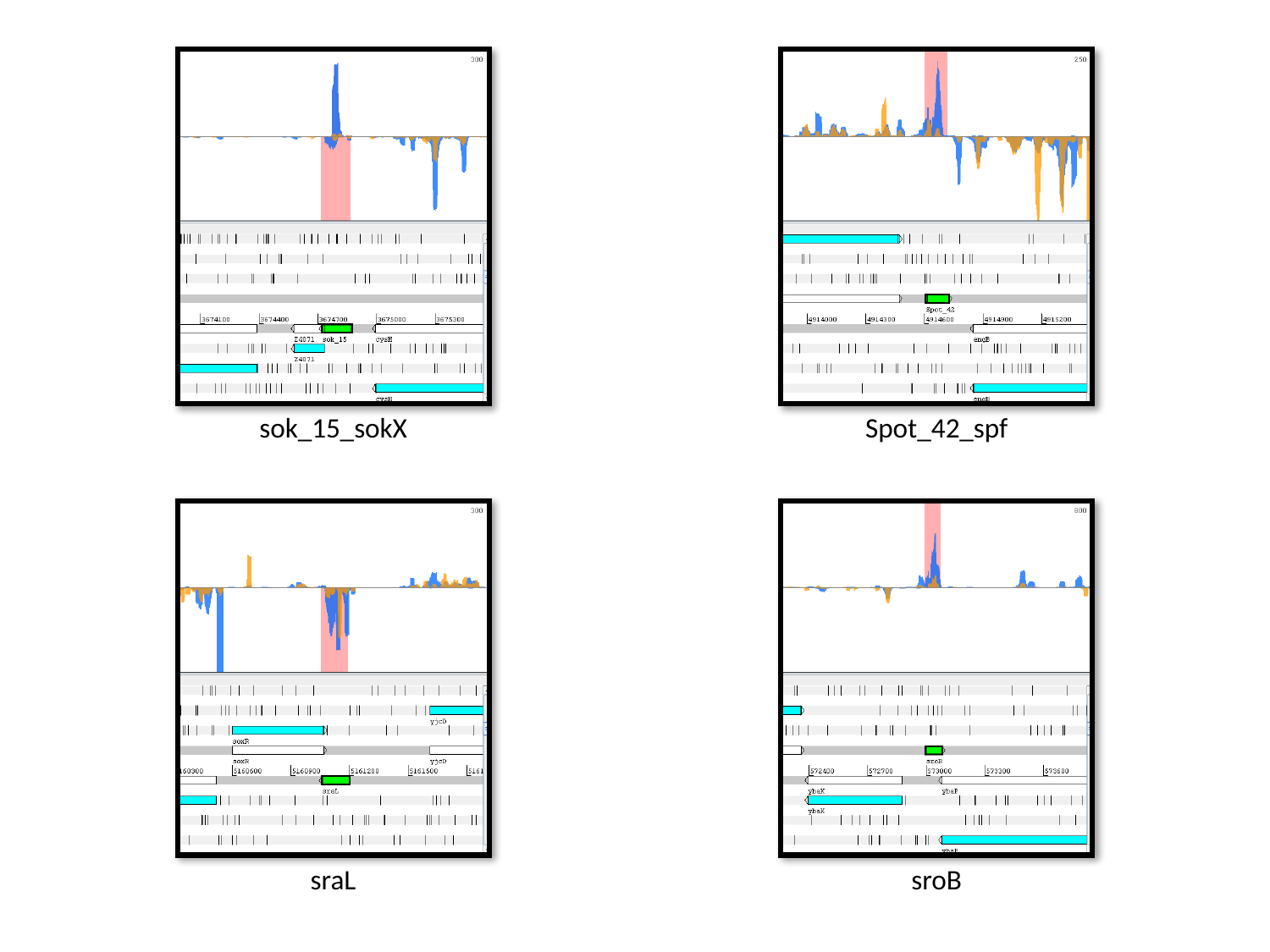

sok_15_sokX
Spot_42_spf
sraL
sroB

## Slide 10
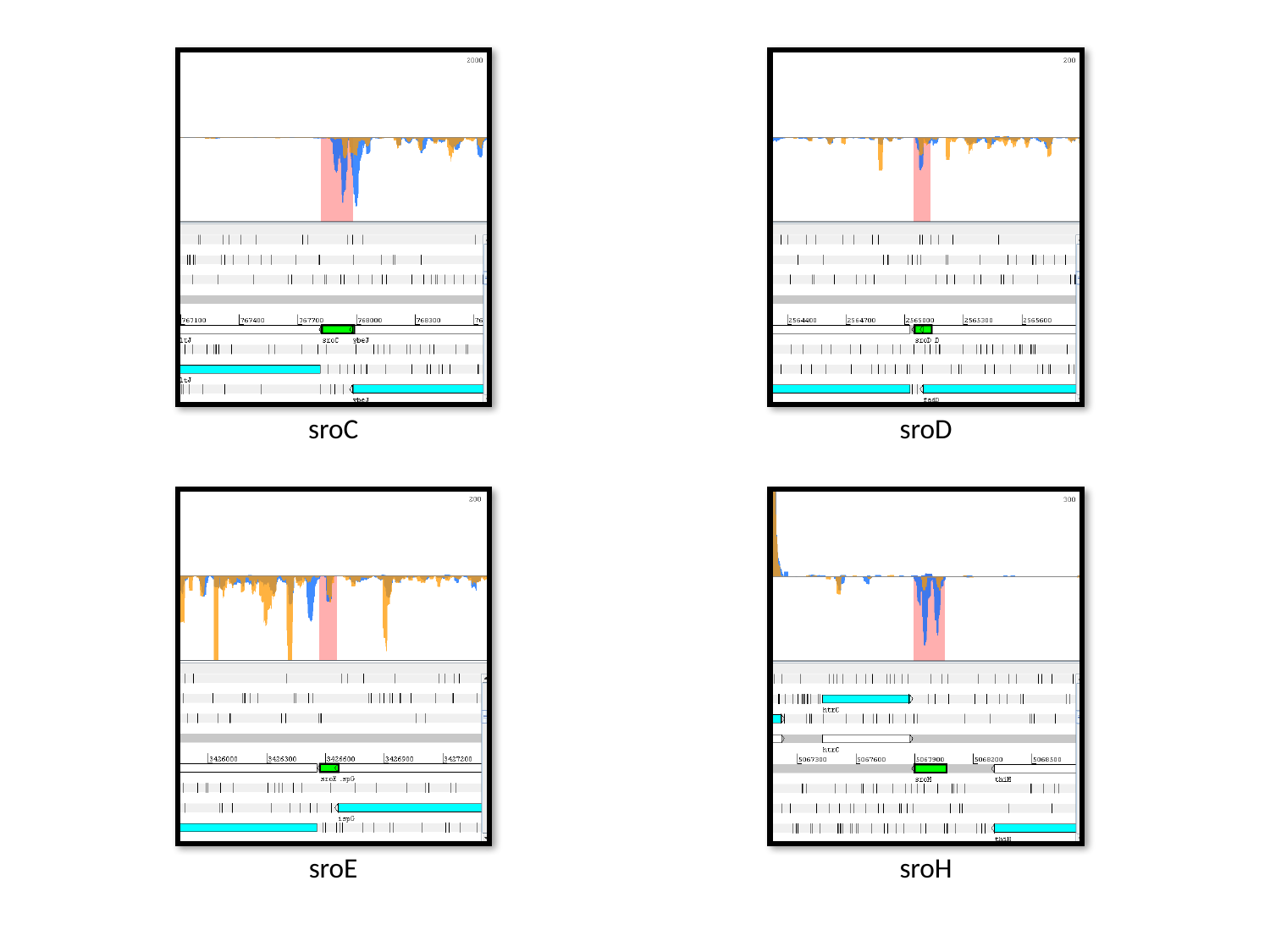

sroC
sroD
sroE
sroH

## Slide 11
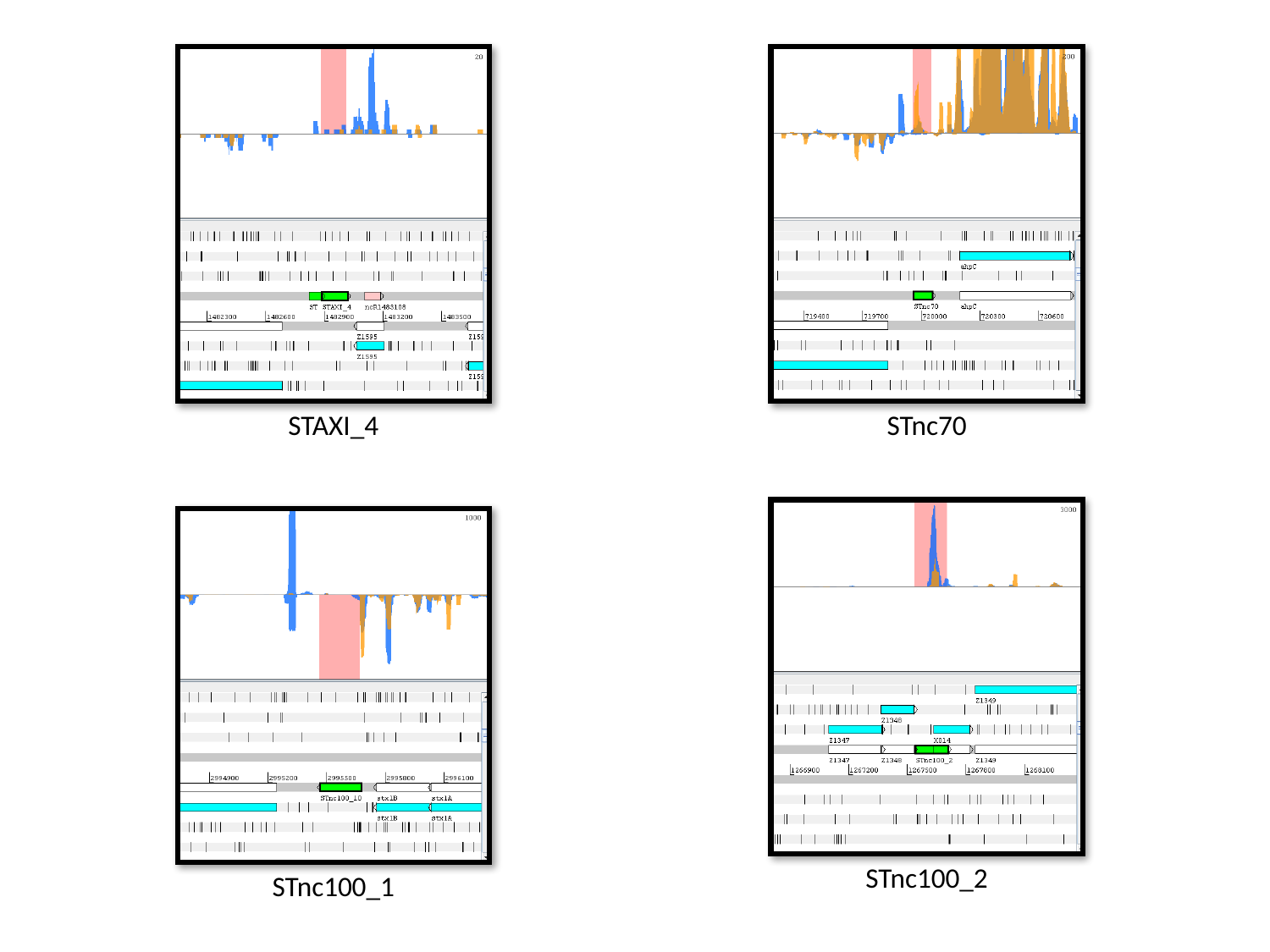

STAXI_4
STnc70
STnc100_2
STnc100_1

## Slide 12
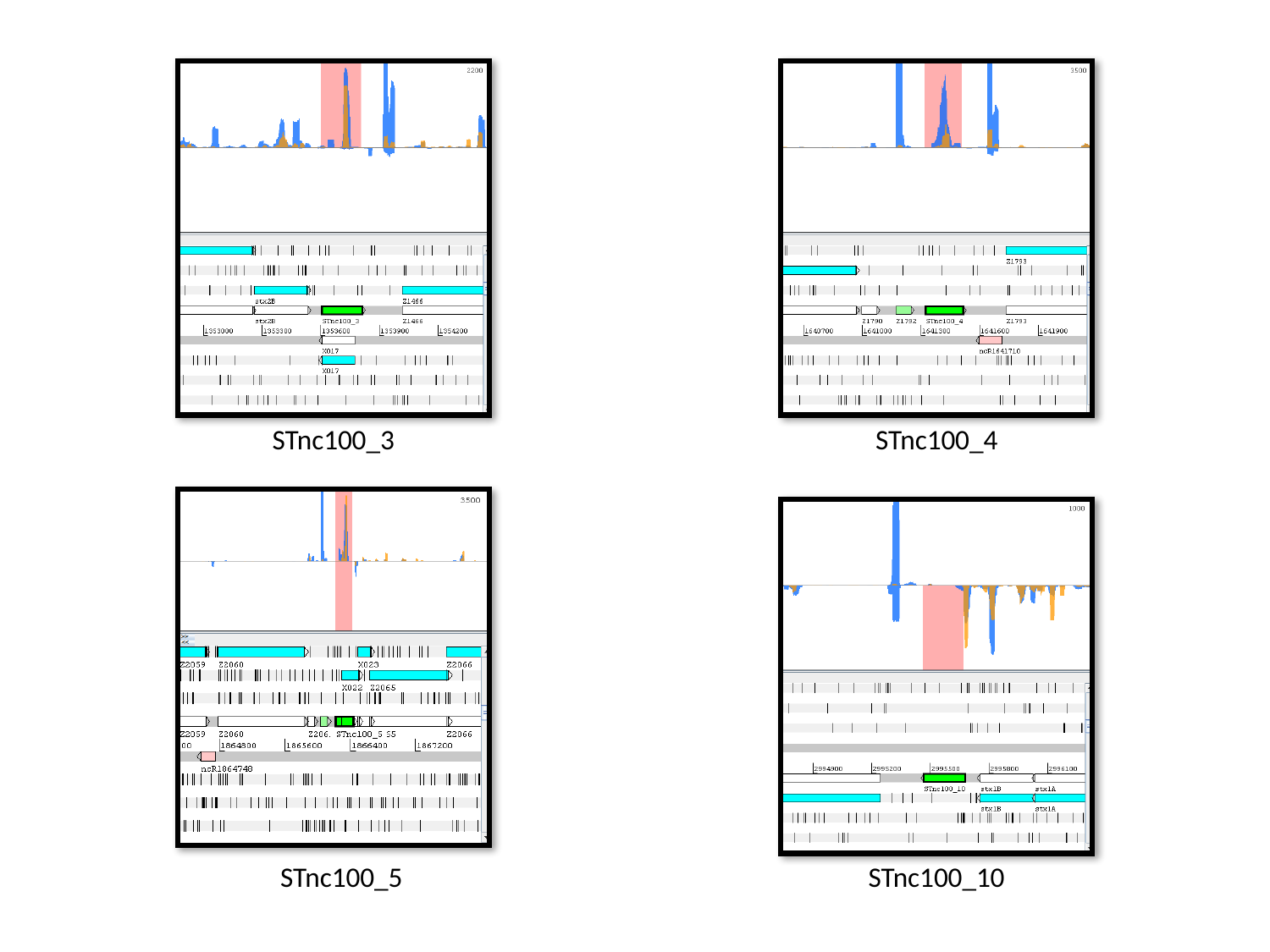

STnc100_3
STnc100_4
STnc100_10
STnc100_5

## Slide 13
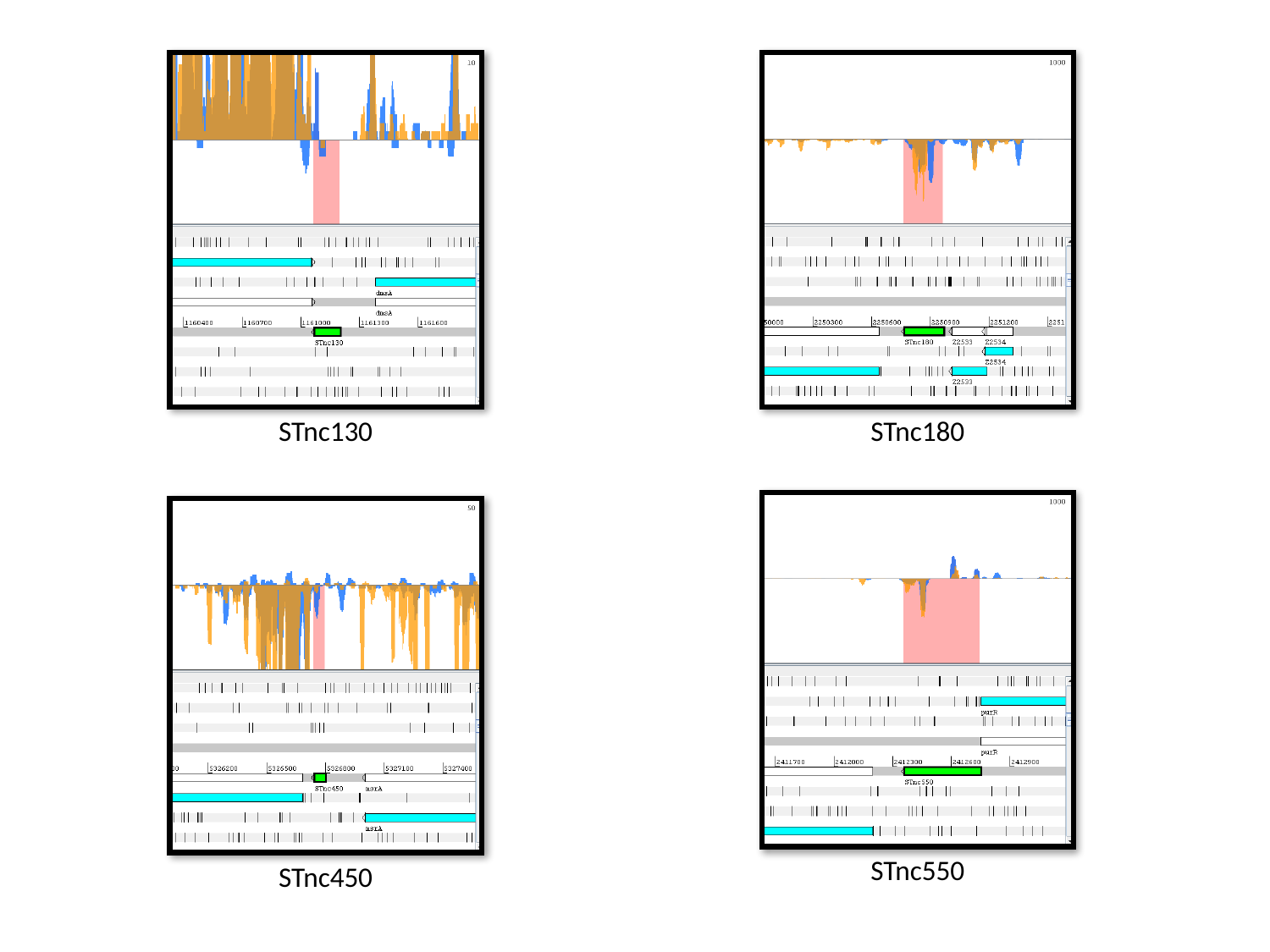

STnc130
STnc180
STnc550
STnc450

## Slide 14
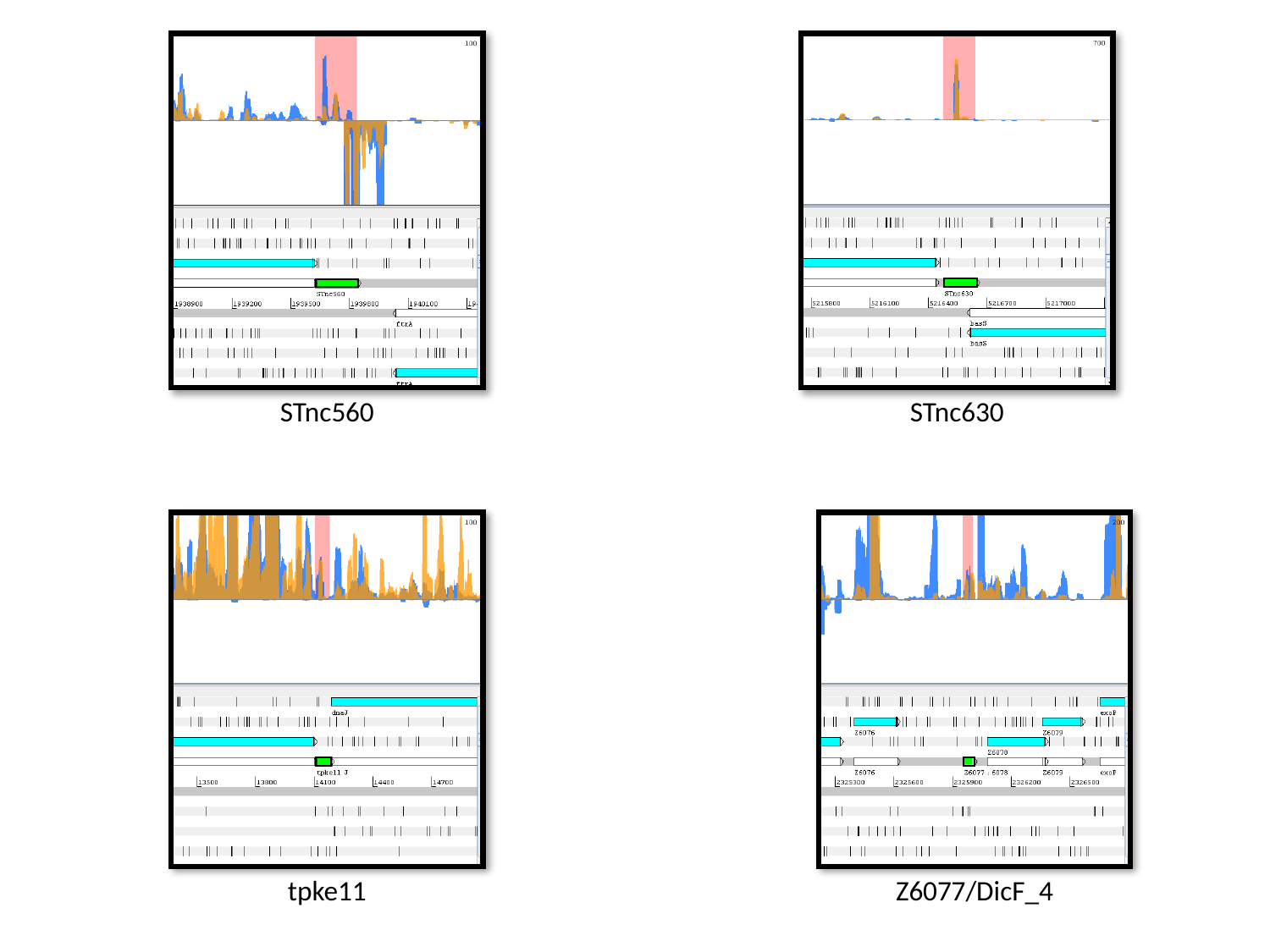

STnc560
STnc630
tpke11
Z6077/DicF_4
